# The genomic tool-kit of the truffle *Tuber melanosporum* programmed cell death

**DOI:** 10.1038/s41420-017-0019-0

**Published:** 2018-02-20

**Authors:** Osvaldo Zarivi, Patrizia Cesare, Anna Maria Poma, Sabrina Colafarina, Antonella Bonfigli, Annegret Kohler, Pierpaolo Aimola, Anna Maria Ragnelli, Giovanni Pacioni, Michele Miranda

**Affiliations:** 1 Department of Life, Health and Environmental Sciences, University of L’Aquila, L’Aquila, 67100 Italy; 2 INRA, UMR INRA-Université de Lorraine ‘Interactions Arbres/Microorganismes’, Laboratoire d’Excellence ARBRE, INRA-Nancy, Champenoux, 54280 France

## Abstract

A survey of the truffle *Tuber melanosporum* genome has shown the presence of 67 programmed cell death (PCD)-related genes. The 67 genes are all expressed during fruit body (FB) development of *T. melanosporum* development; their expression has been detected by DNA microarrays and qPCR. A set of 14 PCD-related genes have been chosen, those with the highest identities to the homologs of other species, for a deeper investigation. That PCD occurs during *T. melanosporum* development has been demonstrated by the TUNEL reaction and transmission electron microscopy. The findings of this work, in addition to the discovery of PCD-related genes in the *T. melanosporum* genome and their expression during the differentiation and development of the FB, would suggest that one of the PCD subroutines, maybe autophagy, is involved in the FB ripening, i.e., sporogenesis.

## Introduction

The genomic tool-kit for the programmed cell death (PCD) has been investigated in the unicellular ascomycete *Saccharomyces cerevisiae* Meyen as a paradigmatic model of eukaryotic cells^1^.

*Tuber melanosporum* Vittad. is a multicellular ascomycete whose genome has been sequenced and studied^2^; however, not yet its PCD genomic tool-kit has been described or some of the possible roles that it may have in the differentiation of reproductive organs, tissues, and cells (the fruiting body FB, the fertile veins, and the spores).

Cells of all living organisms are programmed to self-destruct under certain conditions.

PCD was first described in multicellular metazoans as a developmental strategy whereby unwanted cells are removed to make way for new cellular remodeling and differentiation^3–7^.

Considerable differences exist in the molecular components, regulation, and the role of the apoptotic machinery in different living systems^8,9^.

Such variability is expected in light of the ancient origin of the core apoptotic machinery and the extensive modifications that took place during the evolution of apoptotic networks.

Importantly, however, during the past decade, evidence of PCD has been obtained in both the unicellular fungus, the yeast *Saccharomyces cerevisiae*^1^ and some filamentous fungi^9^, organisms consisting of networks of tubular and multinuclear cells (hyphae), which might or might not be subdivided by cell septa. In these systems, PCD is involved in different biological processes, including interactions with other systems, development, and aging^10^. Some of these processes exhibit typical characteristics of apoptosis, which is one specific type of PCD: externalization of phosphatidylserine, release of cytochrome *c*, involvement of cysteine proteases, the presence of mitochondrial-signaling pathways via homologs of the human apoptosis-inducing factor (AIF)^11^. Apoptotic-like cell death was first described in *Saccharomyces cerevisiae* over 10 years ago, but yeast apoptosis remained controversial, mainly due to its questionable physiological relevance and a lack of molecular and genomic data^12,13^.

Later studies, including the identification and analysis of homologs of apoptotic genes, confirmed the existence of apoptotic-like cell death in fungi^14^. These studies also showed the connection between apoptotic-like cell death and important biological processes such as development, aging, stress responses, and pathogenesis. The emerging role of apoptosis as a key regulator of fungal development suggests that it might be possible to develop new means of controlling fungal infections through manipulation of apoptosis.

However, apoptosis has been described and studied in only a few fungal species, and although homologs of apoptotic genes can be identified in all fungal genomes, to date only a handful of genes have been functionally analyzed. Further research is needed to identify the molecular components and cellular mechanisms controlling apoptosis in fungi. Recognition of the importance of apoptosis for fungal development has led to increased interest and more intense research in recent years, which provide information on various aspects related to fungal apoptosis^15–17^.

The aims of the present work are the investigations on: a) the PCD-related genetic tool-kit of *T. melanosporum*; b) the homologies of *T. melanosporum* PCD-related genes to those of other ascomycetes and species included the human one; c) the involvement of PCD in the differentiation of *T. melanosporum* reproductive system structures.

## Results

From a search within *T. melanosporum* genome^2^ a set of 67 genes involved in PCD has been found (Table S1). In Table S1 the functions of the genes reported are described; some of them not yet annotated while the others, the majority, annotated. The genes found in *T. melanosporum* genome have roles in the different subroutines of PCD (apoptosis, autophagy, necrosis).

In the Table S1 the identity percentages of homology of *T. melanosporum* PCD genes with those of other ascomycetes (*Tuber borchii, Botritis cinerea, Aspergillus nidulans, Neurospora crassa, Magnaporthe grisea, Saccharomyces cerevisiae*) and the human ones are presented. In Table S1 the maximal identities found among *T. melanosporum* PCD genes compared to the homologous of the other ascomycetes reported in Table S1 are shown.

In the Table S1 the reviewed identities of the ascomycetes investigated to the human homologous ones are also reported.

The expressions of the PCD-related genes by DNA microarrays at different developmental stages of *T. melanosporum* FBs are shown in Table S2 and Figure S1.

**Fig. 1:**
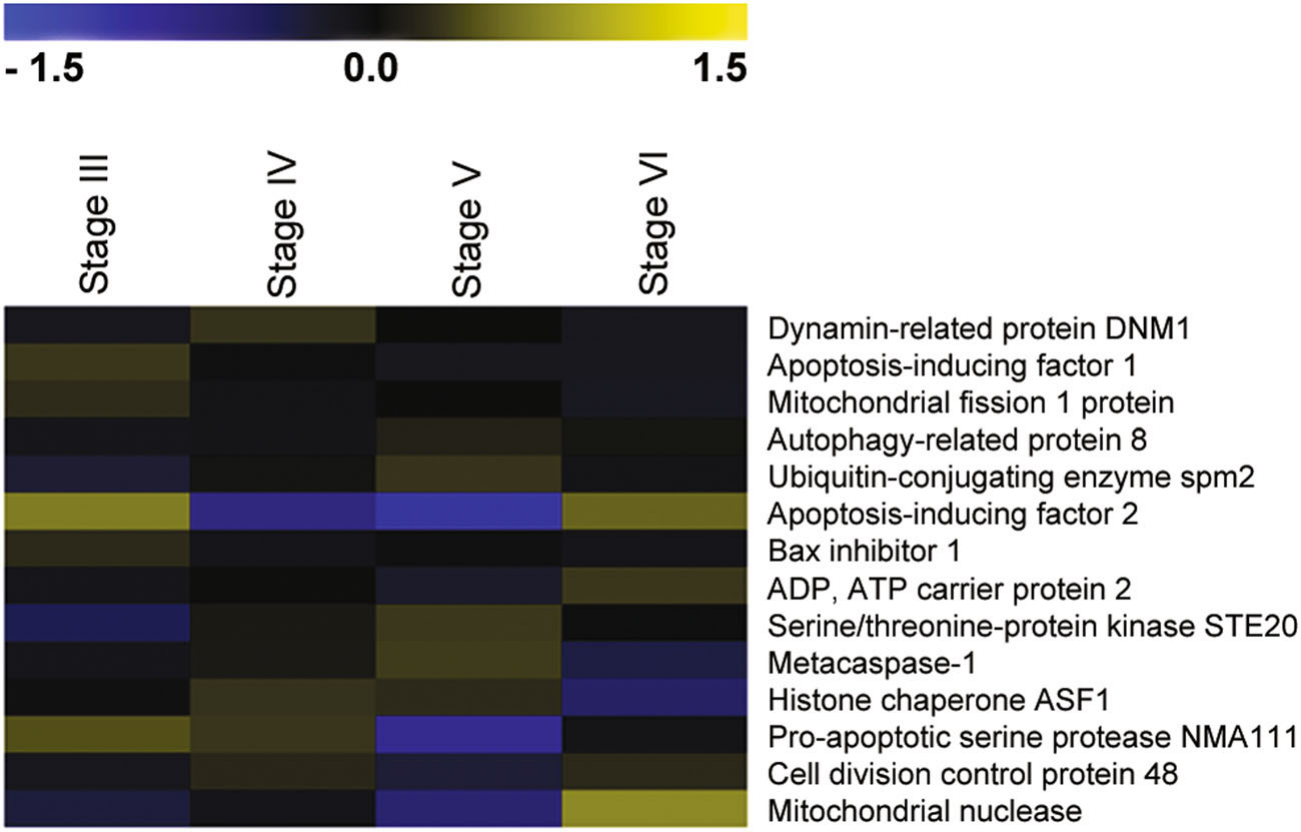
Expression of 14 *Tuber* genes related to programmed cell death in various stages (III–VI) of fruit body development. Heatmap of log2 arbitrary expression values. Relative expression indexes (REI) were calculated for the dataset. For each gene, a mean expression level was calculated from the four samples, and the REI corresponds to the ratio between the expression level measured for a given sample and the mean reference. Log2 transformed data were subjected to MeV software for visualization. Each gene is represented by a row of colored boxes (corresponding to REI values) and a single column represents each stage

From the set of PCD involved genes shown in Table S1, 14 genes with the highest homologies were chosen in order to investigate their expressions by qPCR, in different developmental stages of *T. melanosporum* (Table 1) and for a preliminary gene expression investigation by DNA microarrays (Fig. 1). Most of those genes are involved in apoptosis, some in autophagy. Some of the genes code for PCD promoting proteins (PET9, DNM1, NMA111, aif1, Aifm2, NUC1, STE20, YCA1, CDC48), others for PCD inhibiting proteins (BXI1, FIS1, MMS2), and ASF1 that codes for a chaperone protein involved in nucleosomes assembly and disassembly.

**Table 1:** The fourteen genes having the roles reported in the Table were chosen among the genes involved in PCD present in the *T. melanosporum* genome.

Gene name	Protein	Biological function/process	*Tuber* gene models *Tuber melanosporum*	Blast/identities *Saccharomyces cerevisiae*	Reviewed Max ident	Human
BXI1	Bax inhibitor 1	Protein involved in apoptosis; described as containing a BCL-2 homology (BH3) domain or as a member of the BAX inhibitor family; reported to promote apoptosis under some conditions and to inhibit it in others; translocates to mitochondria under apoptosis-inducing conditions in a process involving Mir1p and Cor1p	GSTUMT00004899001	YNL305C (34%)	LFG4_HUMAN (42.86%)	LFG4_HUMAN (42.86%)
PET9	ADP, ATP carrier protein 2	Catalyzes the exchange of ADP and ATP across the mitochondrial inner membrane	GSTUMT00005644001 (TmelADT)	YBL030C (79%)	ADT_NEUCR (87.00%)	ADT4_HUMAN (53.00%)
FIS1	Mitochondrial fission 1 protein	Fis1 inhibits Dnm1- and Mdv1-mediated mitochondrial fission and cell death, indicating a prosurvival function for Fis1 and a proapoptotic function for Dnm1 and Mdv1 during cell death	GSTUMT00002150001	YIL065C (45%)	FIS1_TUBBO (96.00%)	FIS1_HUMAN (34.46%)
DNM1	Dynamin-related protein DNM1	Microtubule-associated force-producing protein that participates mitochondrial fission	GSTUMT00001624001	YLL001W (67%)	DNM1_YEAST (67.00%)	DNM1L_HUMAN (52.01%)
NMA111	Proapoptotic serine protease NMA111	Nuclear serine protease which mediates apoptosis through proteolysis of the apoptotic inhibitor BIR1	GSTUMT00008284001	YNL123W (51%)	NM111_ASPOR (68.89%)	HTRA2_HUMAN (23.42%)
aif1	Apoptosis-inducing factor 1	Putative FAD-dependent oxidoreductase. Translocates from mitochondria to the nucleus under apoptotic conditions, where it degrades DNA and induces apoptosis	GSTUMT00001651001	AIF1_YEAST (24.81%)	AIF1_SCHPO (41.67%)	AIFM3_HUMAN (35.21%)
Aifm2	Apoptosis-inducing factor 2	Probable oxidoreductase that acts as a caspase-independent mitochondrial effector of apoptotic cell death	GSTUMT00004637001	AIF1_YEAST (24.23%)	AIFM2_MOUSE (27.40%)	AIFM2_HUMAN (26.84%)
NUC1	Mitochondrial nuclease	Major mitochondrial nuclease, has roles in mitochondrial recombination, apoptosis and maintenance of polyploidy	GSTUMT00010203001	YJL208C (60%)	NUC1_YEAST (60.00%)	NUCG_HUMAN (39.32%)
STE20	Serine/threonine-protein kinase STE20	MAP4K component of the MAPK pathway required for the mating pheromone response, haploid invasive growth and diploid pseudohyphal development. Upon exposure to an apoptotic stimulus, the histone deacetylase Hos3p deacetylates K11 and allows Ste20p to phosphorylate S10, required for apoptotic cell death	GSTUMT00006969001 (TmelSte20)	YHL007C (49%)	STE20_TALMA (56.38%)	STK4_HUMAN (40.75%)
YCA1	Metacaspase-1	Mediates cell death (apoptosis) triggered by oxygen stress, salt stress, or chronological aging. Promotes the removal of insoluble protein aggregates during normal growth	GSTUMT00007513001	YOR197W (57%)	MCA1A_ASPTN (69.55%)	CASP7_HUMAN (22.55%)
CDC48	Cell division control protein 48	Involved in spindle disassembly, degradation of ubiquitinated proteins and protein export from the endoplasmic reticulum to the cytoplasm	GSTUMT00010158001	YDL126C (75%)	CDC48_EMENI (86.40%)	TERA_HUMAN (72.86%)
ATG8	Autophagy-related protein 8	Ubiquitin-like modifier involved in cytoplasm to vacuole transport (Cvt) vesicles and autophagosomes formation. With ATG4, mediates the delivery of the vesicles and autophagosomes to the vacuole via the microtubule cytoskeleton	GSTUMT00002234001 (TmelAUT7)	YBL078C (78%)	ATG8_ASPOR (97.46%)	GBRL2_HUMAN (58.97%)
MMS2	Ubiquitin-conjugating enzyme spm2	Has a role in the DNA error-free postreplication repair (PRR) pathway. Lacks catalytic activity by itself. The UBC13/MMS2 heterodimer catalyzes the synthesis of non-canonical poly-ubiquitin chains that are linked through ‘Lys-63’	GSTUMT00003288001	YGL087C (62%)	MMS2_SCHPO (63.77%)	UB2V1_HUMAN (47.48%)
ASF1	Histone chaperone ASF1	Histone chaperone that facilitates histone deposition and histone exchange and removal during nucleosome assembly and disassembly	GSTUMT00007519001	YJL115W (51.69%)	ASF1_EMENI (63.70%)	ASF1A_HUMAN (45.13%)

The choice is based on the highest identities with PCD genes of other ascomycetes and organisms

**Fig. 2:**
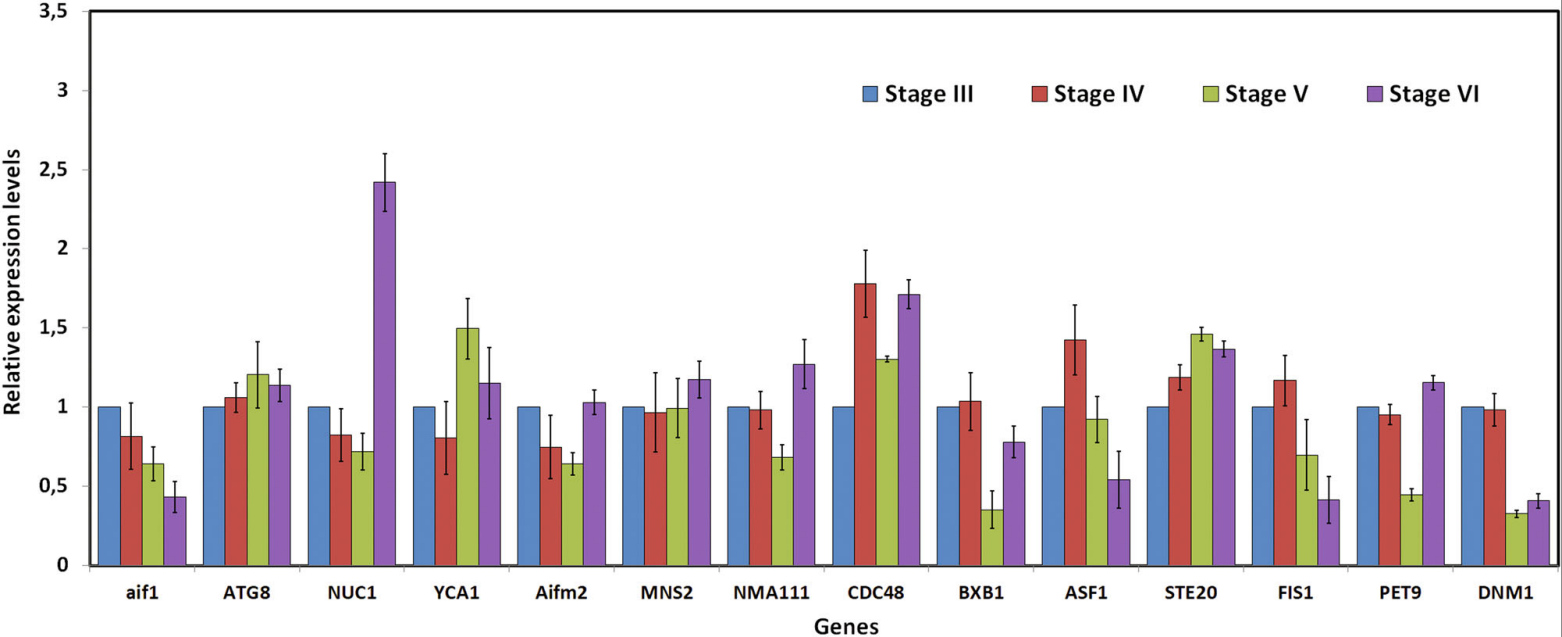
The expressions, as measured by qPCR, of the 14 chosen genes from *T. melanosporum* genome, relative to the expressions at the developmental stage 3. The expression at the developmental stage 3 is assumed as 1 (100%)

Figure 2 shows the relative expressions of the 14 chosen genes, assuming as 1 (100%) their expressions at the *T. melanosporum* developmental stage 3, measured by qPCR.

A strong expression increase of NUC1 occurs at developmental stage 6 while an increase of YCA1 is evident from stages 5 and 6.

CDC48 and STE20 expressions, relative to the developmental stage 3, increase up to the stage 6.

The proapoptotic role of the mitochondrial nuclease NUC1 is well known^18^; *T. melanosporum* mitochondria at stage 6, ripe FB, have been shown being mitochondrial relics as those of yeast under anaerobic life conditions^19^.

Interestingly the metacaspase-1 (YCA1) gene, involved in apoptosis regulation, increases its expression at stages 5 and 6 (Fig. 2) when, as reported later in this work, evident signs of PCD are present within *T. melanosporum* FB.

**Fig. 3:**
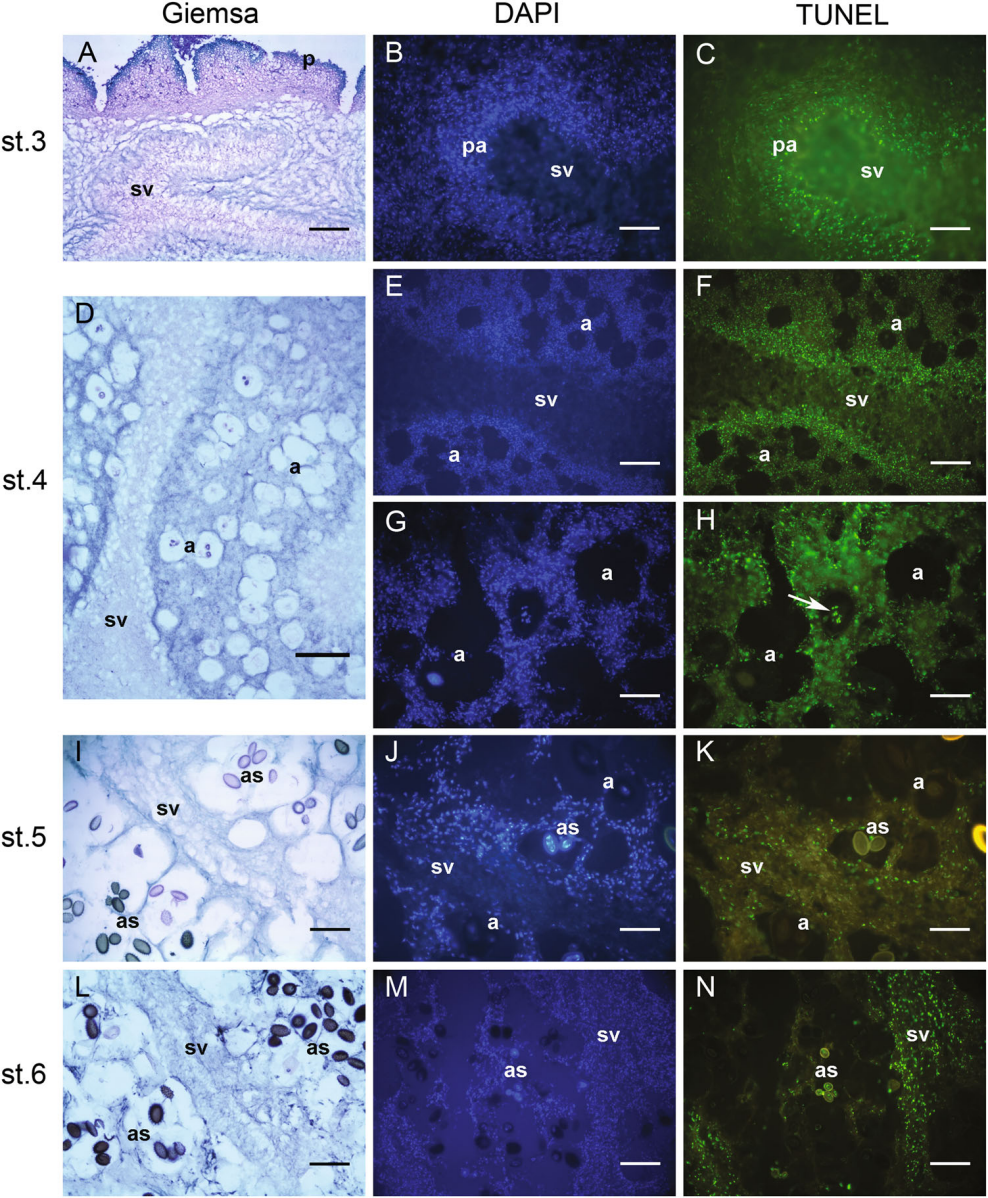
In situ cell death detection by means of TUNEL reaction on cryostat sections of *T. melanosporum* fruit bodies at different developmental stages, Stage 3 (**a**–**c**); stage 4 (**d**–**h**); stage 5 (**i**–**k**); stage 6 (**l**–**n**). The three columns illustrate representative images of, respectively, Giemsa staining (for structural analysis and stage assessment), DAPI staining (for total nuclei visualization), and TUNEL reaction. a asci, as ascospores, p peridium, pa paraphyses, sv sterile vein. Bars = 50 µm in **b**, **c**, **g**, **h**, **j**, and **k**; 100 µm in **e**, **f**, **l**, **m**, and **n**; 200 µm in **a**, **d**, and **i**

**Fig. 4:**
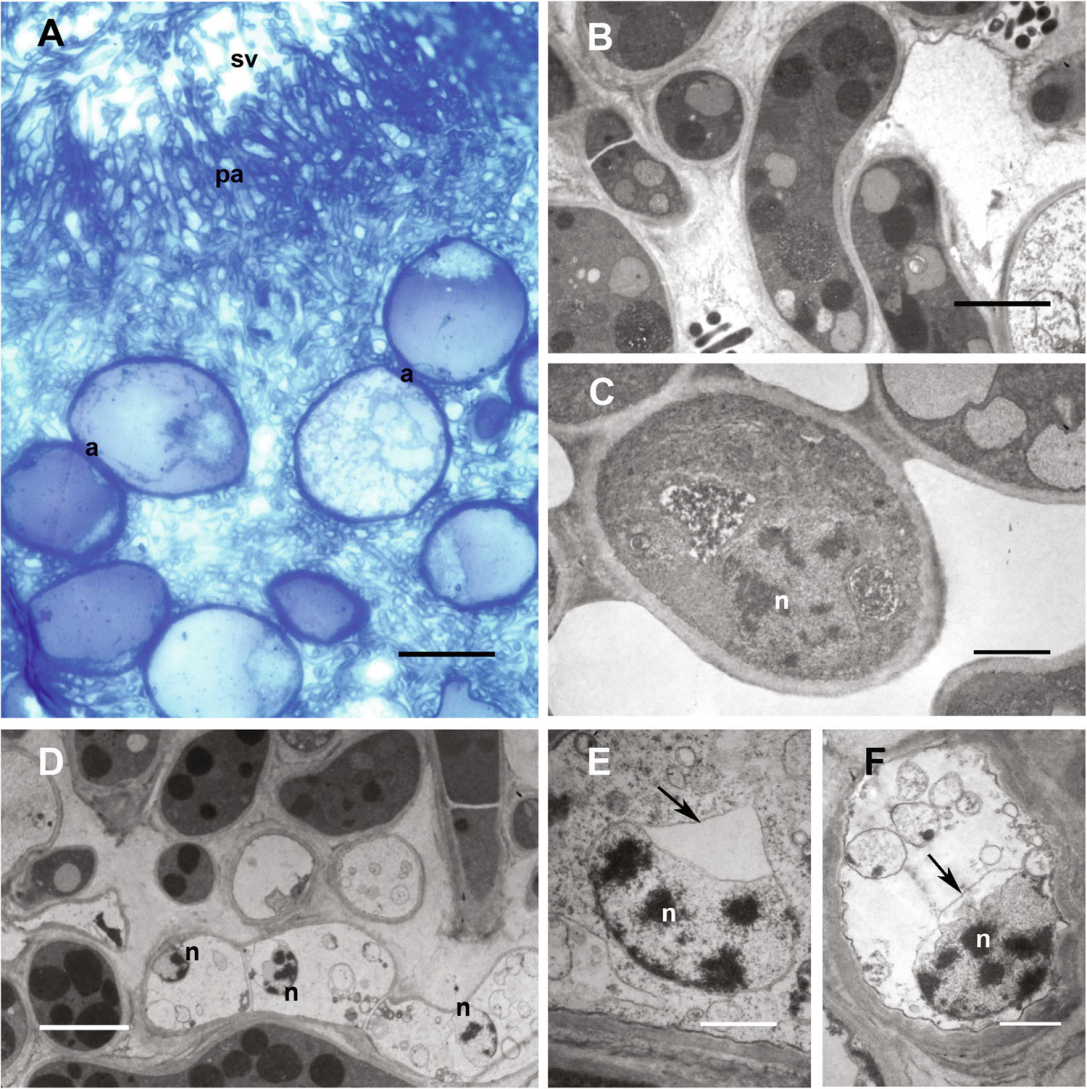
TEM analysis of cell death in *T. melanosporum* (stage 4) fruiting bodies. **a** Toluidine blue-stained semithin section, showing the paraphyses region at the interface between sterile and fertile veins, where significant TUNEL positivity has been detected. **b**, **c** Examples of intact sterile hyphae, with normal nuclei; **d** sterile hyphae showing cytoplasmic emptying and vacuolization. **e**,** f** Altered nuclei with irregular condensed chromatin masses and dilated nuclear envelope lumen (arrow). a asci, n nuclei, pa paraphyses, sv sterile vein. Bars = 50 µm in **a**; 4 µm in **b**, **d**; 2 µm in **c**; 1 µm in **e**, **f**. TEM transmission electron microscopy

The increases of CDC48 and STE20 expressions up to stages 5 and 6 are in line with the role that the proteins coded by these genes have in the control of cell division and the regulation of sexual differentiation, respectively, during the FB ripening, when mitosis and meiosis occur during asci maturation and sporogenesis. The expression of aif1, FIS1, and DNM1, which steadily decreases from the developmental stage 3 up to stage 6, as found by histochemical and transmission electron microscopy (TEM) investigations (terminal deoxynucleotidyl transferase dUTP nick end labeling (TUNEL), Fig. 3 and TEM, Fig. 4), signs of PCD, would suggest that TUNEL positivity, in some cells of the *T. melanosporum* FB, is not due to the apoptotic subroutine of PCD but another one (autophagy or necrosis). However a large genetic tool-kit related to PCD is present in the *T. melanosporum* genome; some of the PCD genes are homologous to PCD genes of the human genome and of species other than Ascomycetes, such as mouse, Aves, Amphibia, Zebrafish, Ascidiacea, Mollusca, etc. thus vertebrates and invertebrates (Figure S2). In Figure S2, the deduced AA sequences identities of PCD involved genes of *T. melanosporum* genome versus the human homologous sequences are reported. Figure S2 shows also the percentual identities of the deduced AA sequences of various invertebrates and vertebrates versus the human homologous sequences.

**Fig. 5:**
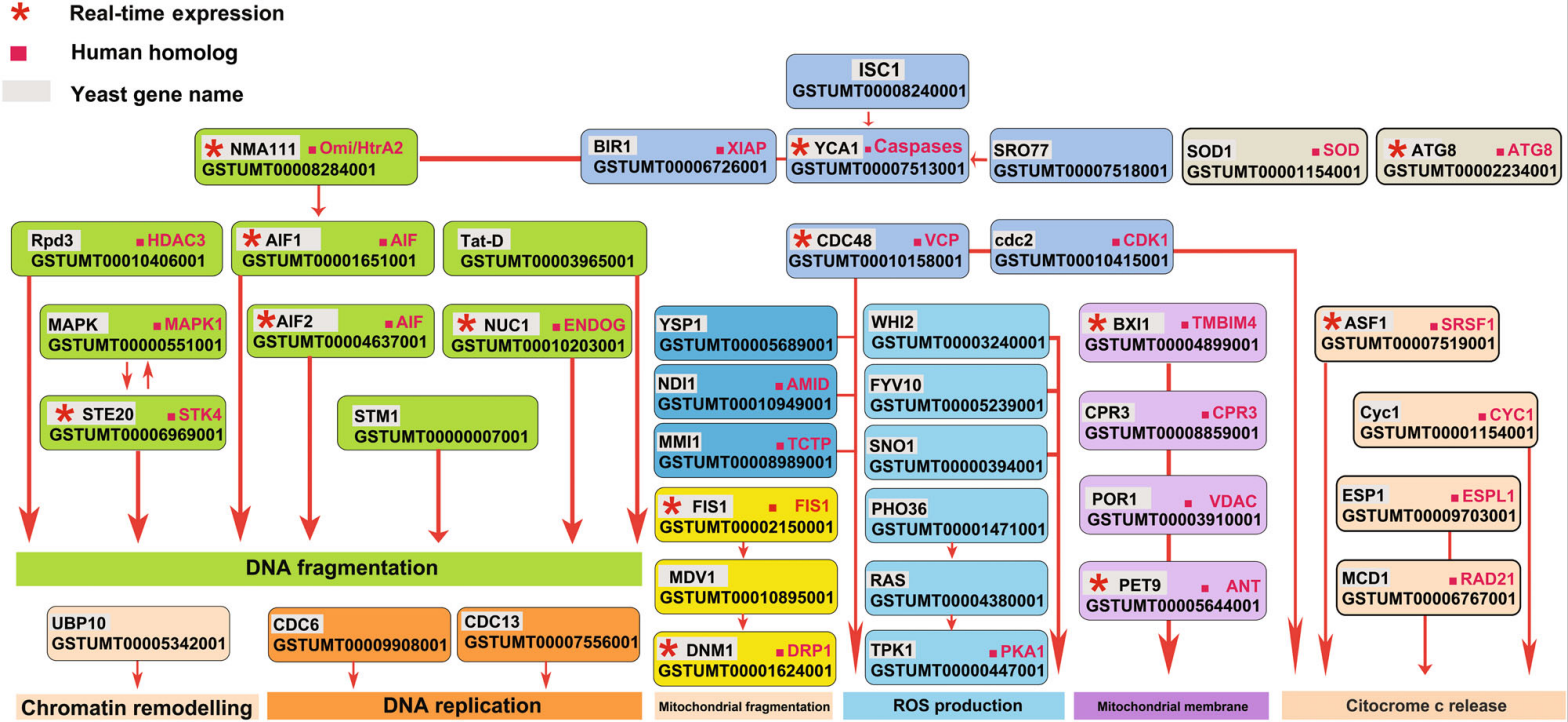
**40** ***T. melanosporum*** **PCD-related genes, with their human and yeast homologs, and the roles they have in programmed cell death.**

The key players of *T. melanosporum* PCD, the yeast, and human homologs and the roles they have in PCD subroutines are reported in Fig. 5. It is evident from the Fig. 5 that *T. melanosporum* has the genetic tool-kit to perform PCD. Figure 3 shows the TUNEL assay positivity of *T. melanosporum* FBs at different developmental stages.

PCD phenomena in *T. melanosporum* FBs at different developmental stages were assessed by means of the TUNEL reaction, which can reveal the presence of nuclei with fragmented DNA through terminal deoxynucleotidyl transferase (TdT)-mediated labeling of free 3′-OH extremities of DNA breaks. The same sections used for the TUNEL reaction were subsequently stained with 4′,6-diamidino-2- phenylindole (DAPI) to visualize all of the nuclei and so evaluate the extension of the cell death.

Stage 1 (“hyphal stage”) and 2 (“peridial stage”) FBs did not exhibit any significant positivity to the TUNEL reaction (not shown). In stage 3 (“veined stage”) FBs, on the other hand, some TUNEL-positive nuclei could be detected, particularly at the level of the interface between developing sterile and fertile veins, where specialized hyphae called paraphyses begin to appear (Fig. 3a–c). At stage 4 (“ascal stage”) more numerous TUNEL-positive nuclei were visible in the paraphyses (Fig. 3d–f), while (some) TUNEL positivity could be seen also in fertile veins and in some developing asci (Fig. 3g, h). In stage 5 (“sporal stage”) FBs TUNEL-positive nuclei were present both in sterile and fertile veins; in these latter, more specifically, they were particularly evident in some asci-surrounding hyphae (Fig. 3i–k) and, to some extent, at the level of the hypothecium (not shown). Finally, numerous TUNEL-positive nuclei were observed in stage 6 (“pigmented stage”) FBs, mostly at the interface between fertile veins and remnants of sterile veins (Fig. 3l–n).

For TEM analysis, toluidine blue-stained semithin sections were preliminarily observed for general morphological examination and as survey sections to localize the areas of the samples for ultrathin sectioning. We focused in particular on the region at the interface between sterile and fertile veins, where paraphyses are organized in a palisade delimiting the sterile vein and where significant TUNEL positivity had been detected (Fig. 4a); in this zone we could observe the presence of altered nuclei (Fig. 4d–f), with irregular condensed chromatin masses reminding those found sometimes in (animal) apoptotic cells, within sterile hyphae showing cytoplasmic emptying and vacuolization (Fig. 4d). Another remarkable feature observed in these altered nuclei was the recurrent presence of a (generally) single, fairly large dilation of the nuclear envelope lumen (Fig. 4e, f).

## Discussion

The expression during FB development of the 67 genes, related to PCD, found in the *T. melanosporum* genome was preliminarily investigated by DNA microarrays (Table S2, Figure S1 and Fig. 1). Fourteen PCD-related genes, on the base of the highest identities to the homologs of other species, were chosen from the 67 PCD-related genes found and their expression tested too by qPCR to strengthen the microarrays data.

Thus both microarrays and qPCR show that PCD-related genes are expressed during *T. melanosporum* FB development.

The TUNEL reaction positivity and TEM support that PCD occurs too in *T. melanosporum* as well as in other ascomycetes^20^. The autophagy subroutine of PCD in the ascomycete *S. cerevisiae* is associated with differentiation, development (e.g. reproduction and spore germination), and stress responses^21^. Several autophagy-related genes in addition to ATG8, whose expression has been investigated both by qPCR and microarray, occur and are expressed during *T. melanosporum* development and differentiation (Table S2, Figure S1 and Fig. 1). The involvement of autophagy in stress responses, for example, to reactive oxygen species (ROS)-generating processes, such as melanin synthesis might be of some interest in the case of *T. melanosporum* FB development.

Melanin synthesis is important to fungal development and differentiation^19,22^; however, the processes potentially genotoxic and cytotoxic due to the generation of semiquinones, quinones, and ROS by the oxidation of polyphenols by tyrosinase (EC 1.14.18.1)^23^^,24^; ROS are known as trigger of PCD^25^. During *T. melanosporum* development tyrosinase activity steadily increases from the developmental stage 3 to stage 5^26^ so that it is expected an increase of ROS and genotoxic species generation within the FBs, in fact evidences of PCD appear at the developmental stage 5.

In conclusion the aims of this work have been addressed: (a) *T. melanosporum* has a genomic tool-kit related to PCD; (b) the *T. melanosporum* PCD-related genes are homologs to those of other ascomycetes and species included the human one; (c) PCD is involved in the FB development when sterile veins show TUNEL positivity and makes space for the fertile veins (Fig. 4) and also TUNEL positivity appears in some spores. The PCD occurring in the spores of ascomycetes has been previously described^9,20^; however, its role has not yet been made clear; a possible role might be the elimination of spores with damaged genome; may be single cell genomics might address this point and help to a better understanding of the reproductive differentiation of a gastronomic delicacy such as the truffle *T. melanosporum*^27^.

## Materials and methods

### Biological materials

Samples of *T. melanosporum* FBs were collected at different developmental stages in the field near the city of L’Aquila (Italy), using the methods described by Pacioni et al.^28^ in addition to the traditional use of trained dogs. According to the season climatic conditions, in the years of this research, specimen of stage 3 were collected between July and August, of stage 4 in August–September, of stage 5 in September–early November, and of stage 6 in late November to March (four biological replicates for each stage). All of the ascocarps were analyzed morphologically and microscopically and classified by maturation stage according to the criteria defined by Zarivi et al.^29^. For structural analysis and maturation stage assessment, 8–10 µm cryostat sections of each FB were observed after Giemsa staining (1 min).

### TUNEL reaction and DAPI staining

In situ detection of PCD was performed by means of the TUNEL reaction. Simultaneous DAPI staining was used to visualize all of the nuclei.

Cryostat sections (8–10 µm thick) of *T. melanosporum* FBs at different developmental stages were mounted on poly-l-lysine-coated microscope slides and fixed with 4% (w/v) paraformaldehyde in phosphate-buffered saline (PBS) for 20 min at room temperature (RT); then they were washed for 20 min with PBS, dehydrated 2 min in absolute ethanol, and stored at −20 °C until use. Before performing TUNEL reaction, cryopreserved sections were incubated in permeabilization solution (0.1% Triton X-100 in 0.1% Na-citrate) for 2 min at 4 °C. After washing with PBS, the sections were incubated in a humid chamber with complete TUNEL reaction mixture, prepared immediately before use according to the manufacturer’s instructions (“In situ cell death detection kit”; Roche Diagnostics, Mannheim, Germany) for 1 h at 37 °C in the dark. Then they were rinsed three times in PBS and mounted with Vectashield mounting medium (Vector Laboratories, Inc., Burlingame CA, USA) containing DAPI. Positive control sections were permeabilized and treated with DNase I 500 U/ml in 50 mM Tris-HCl buffer, pH 7.5, 10 mM MgCl_2_, 1 mg/ml bovine serum albumin, for 10 min at RT, before the TUNEL reaction. Negative control sections, after permeabilization, were incubated in label solution (without terminal transferase). Observations and photography were performed with a Zeiss Axio Imager.A2 (Jena, Germany) fluorescence microscope (Microscopy Center, University of L’Aquila, Italy) equipped for epi-illumination with appropriate filters.

Fragmented DNA containing nuclei showed yellow-green fluorescence, due to incorporation of fluorescein-dUTP into the DNA strand breakages. Total nuclear DNA could be observed with blue fluorescence of DAPI staining.

### Transmission electron microscopy

For ultrastructural analysis, FB samples were embedded in Durcupan ACM epoxy resin, as follows: the samples were pre-fixed with 3% glutaraldehyde in 0.1 M cacodilate buffer, pH 7.2, for 3 h at room temperature; after washing in the same buffer, they were post-fixed with buffered 1% osmium tetroxide for 2 h at 4 °C, dehydrated in an ethanol series, and embedded in the resin.

Semithin (1 μm) sections were cut with a glass knife using a Sorvall Porter-Blum MT2-B ultramicrotome and stained with 1% toluidine blue in 1% Na-borate. Ultrathin sections (70 nm thick) were stained with 5% uranyl acetate in 70% ethanol and lead citrate (Reynolds) and observed using a Philips CM100 transmission electron microscope (Microscopy Center, University of L’Aquila, Italy).

### Gene selection and primer design

The putative genes encoding proteins involved in PCD were identified at the TuberDB Tuber genome database (http://mycor.nancy.inra.fr/IMGC/TuberGenome/). Searches were also performed using several protein databases (e.g., NPS@:BLAST HomologySearch: https://npsa-prabi.ibcp.fr/cgi-bin/npsa_automat.pl?page=/NPSA/npsa_blast.html; Interpro: https://www.ebi.ac.uk/interpro/; Pfam: http://www.sanger.ac.uk/Software/Pfam/; Prosite: http://www.expasy.org/prosite/; Uniprot: http://www.pir.uniprot.org/; Superfamily: http://supfam.cs.bris.ac.uk/SUPERFAMILY/), as well as genome and EST databases (NCBI: http://www.ncbi.nlm.nih.gov/; Yeast database: http://ycelldeath.com/) were searched for PCD-related sequences in order to probe the Tuber genome database using BLAST algorithms. The detected putative homologs were characterized on the basis of the conserved domains, identities and *E-*values. Further information about the name and structure of the genes was obtained using BLASTP, which is available on NCBI (http://www.ncbi.nlm.nih.gov/) and EMBL (http://www.ebi.ac.uk/Tools/blastall/index.html). Each validated homolog was also used for a BLAST search at http://mycor.nancy.inra.fr/IMGC/TuberGenome/blast.html, which has a database with five reference Ascomycota: *Saccharomyces cerevisiae*, *Neurospora crassa* Shear &B.O. Dodge, *Magnaporthe grisea* (T.T.Hebert)M.E.Barr, *Aspergillus nidulans* (Eidam)G.Winter and *Botrytis cinerea* Pers., and at https://genome.jgi.doe.gov/pages/blast-query.jsf?db=Tubbor1 which has a database with *Tuber borchii* Vittad genome.

A putative homolog in the human genome has also been sought. By analyzing the pathway of yeast, some genes have been identified at key points of the process in order to carry out the real-time PCR analysis. The identified genes are shown in Table S1.

The consensus sequences were used for comparison with genomic sequences, to reveal the exon–intron structure. To facilitate the real-time PCR analysis of all the investigated genes under the same reaction conditions, primers were designed using Primer Express 3.0 software (PE Applied Biosystems, USA) under default parameters. The primers, wherever possible, were designed spanning an intron to detect any genomic DNA contamination. For information on primer sequences see Table 2

**Table 2:** Primers used in this study

Name	Accession number	Primer name	Primer sequence 5'–3'; Tm (°C).	Product (bp)	(%) PCR efficiency
18S rRNA	AM748736.1	18S	F: 5′-CCAATGGAAGTTTGAGGCAATAA-3′ (53.8), R: 5′-CCAATGGTGATGAACTCGTTGA-3′ (55.3)	100	95.03
Elongation factor 1-alpha	GSTUMT00000021001	TEFA	F: 5′-AAGGGTGCCGAGTCTTTCAA-3′ (56.7), R: 5′-TATGAGCGGTGTGGCAGTCA-3′ (58.8)	100	94.57
Glucose-6-phosphate dehydrogenase	GSTUMT00008696001	G6PD	F: 5′-CGCGATGAGAAGGTCAGAGTT-3′ (56.9), R: 5′-CAGGCTTGCTGCCATCAAG-3′ (57.0)	100	97.31
Apoptosis-inducing factor 1	GSTUMT00001651001	AIF1	F: 5′-AGCGACAATCAGAGCTGGTAAAC-3′ (60.6), R: 5′-CCCAGAGCCACCTCCAACTA-3′ (61.4)	100	99.55
Apoptosis-inducing factor 2	GSTUMT00004637001	AIF2	F: 5′-GGGCCGCGAAGAAGATTGTC-3′ (61.4), R: 5′-CGGTACCTGTGGCAGGAGTT-3′(61.4)	137	98.16
Autophagy-related protein 8	GSTUMT00002234001	ATG8	F: 5′-TATGCAGACCGTATTCCCAGTTATTTG-3′ (61.9), R: 5′-AGTCAAGTCCGCAGGAACCA-3′ (59.4)	96	95.44
Mitochondrial nuclease	GSTUMT00010203001	EndoG	F: 5′-GGGGATCGGCATCGGAGCA-3′ (63.1), R: 5′-TGCGGCGGGGACCTGGT-3′ (62.4)	117	99.42
Metacaspase-1, Ca2+-dependent cysteine protease	GSTUMT00007513001	YCA1	F: 5′-CAAAGATGCACAACCCAATGA-3′ (55.9), R: 5′-TCGTACCCATCGCCTTATCAC- 3′ (59.8)	96	98.16
Proapoptotic serine protease NMA111	GSTUMT00008284001	NMA111	F: 5′-GTGGATTATTGAGTCCCTTGACAA-3′ (59.3), R: 5′-AGGTGTGCATGGTGTGAAGGT-3′ (59.8)	137	92.39
Cell division control protein 48	GSTUMT00010158001	CDC48	F: 5′-CCGGATTAGACTTGGCGATGT-3′ (59.8), R: 5′-TCCTTCAACAGTGTCTGCAATAGG-3′ (61.0)	100	97.63
Bax Inhibitor 1	GSTUMT00004899001	BAX1	F: 5′-TGTTCGATACGCAGATGATTATGA-3′ (57.6), R: 5′-CCTCAGGATAGCAAGGAACAAGTTA-3′ (61.3)	107	96.06
Histone chaperone ASF1	GSTUMT00007519001	ASF1	F: 5′-TTTGCCATCAAGTGGGACTCT-3′ (57.9), R: 5′-CTCCGTAATTGTCGGCATCA-3′ (57.3)	100	96.06
Serine/threonine-protein kinase STE20	GSTUMT00006969001	STE20	F: 5′-GCTCGCCAACGCTTTTTCT-3′ (56.7), R: 5′-TGAGTATTCCGATCACCAGCAA-3′ (58.4)	108	96.84
Mitochondrial fission 1 protein	GSTUMT00002150001	FIS1	F: 5′-TTGGCGTCCAGACTAAGTTCAA-3′ (58.4), R: 5′-CGGAAGATATCAGTCAATAACCTAACC-3′ (61.9)	100	99.25
ADP, ATP carrier protein 2	GSTUMT00005644001	PET9	F: 5′-AGAACAATCTACTTTCCTCGTTGACTT-3′ (60.4), R: 5′-GATAAGGAGCTTGATACGCTCAATG-3′ (61.3)	100	97.63
Dynamin-related protein	GSTUMT00001624001	DNM1	F: 5′-AGCTGCAGGATCTTGTCTTTAATACTATT-3′ (61.0), R: 5′-ACCGAGGATTTCCCGCTAGA-3′ (59.4)	100	99.46
Ubiquitin-conjugating enzyme spm2	GSTUMT00003288001	SPM2	F: 5′-CGAGAACCGCATTTACAGTTTG-3′ (58.4), R: 5′-AGGTGCCTTAACGCTGTACCA-3′ (57.6)	100	95.30

Primer sequences, accession numbers, amplicon sizes and PCR efficiencies are indicated

### Isolation of total RNA

Total RNA were extracted from stages 3–6 (100 mg each) using TRIzol_Reagent with the PureLink_RNA Mini Kit (Ambion by Life Technologies Carlsbad, CA, USA). RNase-free mortars and pestles were used in combination with liquid nitrogen to disrupt frozen tissue samples into a powder. One hundred milligrams of powdered tissue samples in 1 mL TRIzol Reagent were homogenized by using a mortar and pestle^26^, and then through three cycles of pestle strokes at 200 riv/min in a potter Helvehjem homogenizer. Samples were centrifuged for 10 min at 12,000 *g* and the supernatant was removed and incubated for 3 min at room temperature. Then 0.2 mL chloroform per 1 mL TRIzol Reagent used were added, the tubes were vigorously shaken by hand for 15 s, incubated at room temperature for 3 min, and were centrifuged at 12,000 *g* for 15 min at 4 °C. The upper colorless phase containing the RNA was transferred to a new Rnase-free tube, adding an equal volume 100% ethanol and vortexing to mix well. Binding, washing, and elution steps were performed using the PureLink_RNA Mini Kit according to the manufacturer’s instructions (Ambion by Life Technologies Carlsbad, CA, USA). Then, the RNA was treated with Recombinant Rnase-free Dnase I from bovine pancreas (Roche Diagnostics, Indianapolis, USA) according to the manufacturer’s instructions. Absence of DNA was checked by comparing cDNA samples with RNA samples which were not reverse transcribed (minus RT control). The purity of all RNA samples was assessed at absorbance ratios of A260/A280 and A260/A230 using a Nanodrop 2000 spectrophotometer (Thermo Scientific, Waltham, WA) and the integrity of the RNA was immediately checked using 1.2% agarose gel electrophoresis (1.3 µg samples). RNA concentration was calculated based on absorbance values at 260 nm, and RNA samples were stored at −80 °C until use.

### cDNA synthesis and quantitative real-time reverse transcription-polymerase chain reaction (RT-PCR)

One microgram of total RNA from each sample was reverse transcribed using the SuperScript III First-Strand Synthesis SuperMix for qRT-PCR (Invitrogen by Life Technologies, Carlsbad, CA, USA), following the manufacturer’s instructions. Quantitative PCR was performed with the SYBR^®^ GreenER™ qPCR SuperMix for ABI PRISM^®^, which is a ready-to-use cocktail containing all components (including ROX Reference Dye at a final concentration of 500 nM), except primers and template. All the PCRs were performed under following conditions: 2 min at 50 °C, 10 min at 95 °C, and 40 cycles of 15 s at 95 °C and 1 min at 60 °C in 96-well optical reaction plates (Applied Biosystems, USA). The specificity of the qRT-PCR reactions was monitored through melting curve analysis (60–95 °C), after 40 cycles, using SDS software (version 1.4; Applied Biosystems), all PCR assays produced a single amplicon of the expected size. The gene-specific amplification efficiency was calculated by linear regression analysis of the standard curve. The sequences of *T. melanosporum* 18S rRNA, Elongation factor 1-alpha, glucose-6-phosphate dehydrogenase, employed as an internal standard^29^, and the sequences of the specific primers used to analyze the expression of the selected genes involved in the metabolism apoptosis, are provided in Supplementary Table 1. Each sample was tested in triplicate by quantitative PCR, and the samples obtained from at least four independent experiments were used to calculate the means and standard error; the results were considered to be significant if *P < *0.05.

### cDNA synthesis and whole-genome oligoarray analysis

Total RNA was isolated from Stage 3–6 FBs. Double-stranded cDNA was synthesized and amplified using the SMART PCR cDNA Synthesis Kit (Ozyme, Saint-Quentin-en-Yvelines, France), according to the manufacturer’s instructions, and used for hybridizations to NimbleGen oligoarrays^2^. Single dye labeling of samples, hybridization procedures, and data acquisition were performed at the NimbleGen facilities (NimbleGen Systems, Reykjavik, Iceland) following their standard protocol. The *T. melanosporum* custom-exon expression array (GPL8982) manufactured by Roche NimbleGen Systems (Madison, WI, USA) contained five independent, non-identical, 60-merprobes per gene model coding sequence. Included in the oligoarray were 7496 annotated protein-coding gene models, 5736 TE sequences, 3913 random 60-mer control probes, and labeling controls. For 1876 gene models, technical duplicates were included in the array.

Microarray probe intensities were quantile normalized across chips and average expression levels were calculated for each gene from the independent probes on the array and were used for further analyses^30^. Raw array data were filtered for non-specific probes (a probe was considered as non-specific if it shared more than 90% homology with a gene model other than the gene model it was made for) and renormalized using the ARRAYSTAR software (DNASTAR, Madison, WI, USA). For 1015 gene models, no reliable probes remained. Expression values are arbitrary units from 1 to 57,000.

The complete expression dataset is available as series (GPL8982) at the Gene Expression Omnibus at NCBI (http://www.ncbi.nlm.nih.gov/geo/).

## Electronic supplementary material

Supplementary figure legends

Supplementary Table S1

Supplementary Table S2

Supplementary Figure S1

Supplementary Figure S2
