# Supplementary material for: The genomic tool-kit of the truffle Tuber melanosporum programmed cell death
Source: Cell Death Discov. 2018 Feb 20;4:32. doi: 10.1038/s41420-017-0019-0 (PMC5841409; doi:10.1038/s41420-017-0019-0)
Supplement: Supplementary file 1 — Supplementary figure legends [file 41420_2017_19_MOESM1_ESM.docx]

Figure S1 Expression of 67 *T. melanosporum* PCD related genes in various stages (III-VI) of FBs. Heatmap of log2 arbitrary expression values. Relative expression indexes (REI) were calculated for the dataset. For each gene, a mean expression level was calculated from the four samples, and the REI corresponds to the ratio between the expression level measured for a given sample and the mean reference. Log2 transformed data were subjected to MeV software for visualization. Each gene is represented by a row of colored boxes (corresponding to REI values) and a single column represents each stage.

Figure S2 Phylogenetic relationship among fungi, invertebrates and vertebrates based on % AA sequence identities with human homologous genes assumed as 100%. Common ancestors are indicated as circles (0).
