# Supplementary material for: The genomic tool-kit of the truffle Tuber melanosporum programmed cell death
Source: Cell Death Discov. 2018 Feb 20;4:32. doi: 10.1038/s41420-017-0019-0 (PMC5841409; doi:10.1038/s41420-017-0019-0)
Supplement: Supplementary file 2 — Supplementary Table S1 [file 41420_2017_19_MOESM2_ESM.docx]

**Table S1** The table shows the *T. melanosporum* PCD involved genes, the AA sequences identities to the homologs of various ascomycetes and human ones and their roles in PCD subroutines.

| **GENE NAME** | **Protein** | **Biological function/process** | **Tuber gene models**  ***Tuber melanosporum*** | **Blast/Identities**  ***Tuber borchii*** | **Blast/Identities**  ***Botrytis***  ***cinerea*** | **Blast/Identities**  ***Aspergillus* *nidulans*** | **Blast/Identities**  ***Neurospora***  ***Crassa*** | **Blast/Identities**  ***Magnaporthe oryzae*** | **Blast/Identities**  ***Saccharomyces cerevisiae*** | **MAX**  **Identies** | **Reviewed**  **Max identities** | **Human/Idendities** |
| --- | --- | --- | --- | --- | --- | --- | --- | --- | --- | --- | --- | --- |
| **BXI1** | Bax inhibitor 1 | Protein involved in apoptosis; described as containing a BCL-2 homology (BH3) domain or as a member of the BAX inhibitor family; reported to promote apoptosis under some conditions and to inhibit it in others; localizes to ER and vacuole; may link the unfolded protein response to apoptosis via regulation of calcium-mediated signaling; translocates to mitochondria under apoptosis-inducing conditions in a process involving Mir1p and Cor1p | GSTUMT00004899001 | Protein ID: 1163386  (80%) | BC1G_09605  (65%) | AN1133  (60%) | NCU02463  (57%) | MGG_03089  (56%) | YNL305C  **BXI1**  (34%) | KFY68939.1  A0A094DRK5 A0A094DRK5_9PEZI  (66%) | Q9HC24 LFG4_HUMAN  **TMBIM4**  (42.86%) | Q9HC24 LFG4_HUMAN  (42.86%) |
| **MIR1** | Mitochondrial phosphate carrier protein | Transport of phosphate groups from the cytosol to the mitochondrial matrix.May play a role regulation of the mitochondrial permeability transition pore (mPTP). | GSTUMT00009609001  TmelMIR1 | Protein ID:989344  (98%) | BC1G_02096  (79%) | AN0870  (85%) | NCU05390  (85%) | MGG_02370  (81%) | YJR077C  **MIR1**  (62%) | XP_018076070.1  A0A132B7X9 A0A132B7X9_9HELO  (84%) | P23641 MPCP_YEAST  **MIR1**  (62.00%) | Q00325 MPCP_HUMAN (40.00%) |
| **PET9** | ADP,ATP carrier protein 2 | Catalyzes the exchange of ADP and ATP across the mitochondrial inner membrane. | GSTUMT00005644001 (TmelADT) | Protein ID: 1029732  (96%) | BC1G_02099  (87%) | AN4064  (87%) | NCU09477  (87%) | MGG_06656  (87%) | YBL030C  **PET9**  (79%) | CCX09646.1  U4L1W8  U4L1W8_PYROM  (92.00%) | P02723  ADT_NEUCR  **aac**  (87.00%) | Q9H0C2  ADT4_HUMAN (53.00%) |
| **POR1** | Mitochondrial outer membrane protein porin 1 | Forms a channel through the cell membrane that allows diffusion of small hydrophilic molecules. | GSTUMT00003910001 | Protein ID: 1195320  (98%) | BC1G_04187  (62%) | AN4402  (70%) | NCU04304  (60.42%) | MGG_00968  (60%) | YNL055C  **POR1**  (46%) | XP_011107087.1  S8CB60 S8CB60_DACHA  (75.00%) | P07144  VDAC_NEUCR  **B7H23.070**  (60.42%) | P21796  VDAC1_HUMAN  (32,74%) |
| **FIS1** | Mitochondrial fission 1 protein | Fis1 inhibits Dnm1- and Mdv1-mediated mitochondrial fission and cell death, indicating a prosurvival function for Fis1 and a proapoptotic function for Dnm1 and Mdv1 during cell death | GSTUMT00002150001 | Protein ID: 1117806  (96%) | BC1G_12585  (77%) | AN6225  (74%) | NCU05313  (74%) | MGG_06075  (72%) | YIL065C **FIS1**  (45%) | Q6WRS2  FIS1_TUBBO  (96.00%) | Q6WRS2  FIS1_TUBBO  **FIS1**  (96.00%) | Q9Y3D6  FIS1_HUMAN  (34,46%) |
| **MDV1** | Mitochondrial division protein 1 | Involved in mitochondrial fission. | GSTUMT00010895001 | Protein ID: 1100822  (96%) | BC1G_07440  (61%) | AN6867  (58%) | NCU07724  (58%) | MGG_01711  (54%) | YJL112W  **MDV1**  (33%) | CCX33353.1  U4LN53 U4LN53_PYROM  (64.57%) | Q1DIW7 MDV1_COCIM  **MDV1**  (62.32%) | ------------------- |
| **DNM1** | Dynamin-related protein DNM1 | Microtubule-associated force-producing protein that participates mitochondrial fission. | GSTUMT00001624001 | Protein ID: 1070102  (93%) | BC1G_02461  (85%) | AN8874  (85%) | NCU09808  (84%) | MGG_06361  (84%) | YLL001W  **DNM1**  (67%) | XP_007583479.1  R1GL64 R1GL64_BOTPV  (79,00%) | P54861 DNM1_YEAST  **DNM1**  (67,00%) | O00429 DNM1L_HUMAN  (52,01%) |
| **BIR1** | Protein bir1 | Seems to act in the pleiotropic control of cell division. May participate in chromosome segregation events | GSTUMT00006726001 | Protein ID: 1118678  (87%) | BC1G_14521  (44%) | AN0715  (37%) | NCU06621  (28%) | MGG_04912  (33%) | P47134  BIR1_YEAST  **BIR1**  (21.39%) | CCX05468.1 U4KVC6 U4KVC6_PYROM  (36%) | O14064 BIR1_SCHPO  **bir1**  (23.56%) | Q96CA5 BIRC7_HUMAN  (19,08%) |
| **NMA111** | Pro-apoptotic serine protease NMA111 | Nuclear serine protease which mediates apoptosis through proteolysis of the apoptotic inhibitor BIR1 | GSTUMT00008284001 | Protein ID: 1098460  (97,31%) | BC1G_15876  (69%) | AN10673  (67%) | NCU05200  (66%) | MGG_01824  (66%) | YNL123W  **NMA111**  (51%) | CCX32263.1  U4LJY6  U4LJY6_PYROM  (78%) | Q2TYB1 NM111_ASPOR  **nma111**  (68.89%) | O43464 HTRA2_HUMAN (23.42%) |
| **aif1** | Apoptosis-inducing factor 1 | Putative FAD-dependent oxidoreductase. Translocates from mitochondria to the nucleus under apoptotic conditions, where it degrades DNA and induces apoptosis | GSTUMT00001651001 | Protein ID: 1133884  (89%) | BC1G_09030  (59%) | AN9103  (55%) | NCU05850  (57%) | MGG_08262  (56%) | P52923  AIF1_YEAST  **AIF1**  (24.81%) | CCX29933.1  U4LDK3 U4LDK3_PYROM  (60%) | Q10499  AIF1_SCHPO  **aif1**  (41,67%) | Q96NN9  AIFM3_HUMAN  (35.21%) |
| **Aifm2** | Apoptosis-inducing factor 2 | Probable oxidoreductase that acts as a caspase-independent mitochondrial effector of apoptotic cell death | GSTUMT00004637001 | Protein Id: 1078336  (87%) | BC1G_13095  (32.79 %) | AN9315  (30%) | NCU09447  (28%) | MGG_06179  (33%) | P52923  AIF1_YEAST  **AIF1**  (24.23%) | CCX04772.1  U4KUL9 U4KUL9_PYROM  (39%) | Q8BUE4  AIFM2_MOUSE  **Aifm2**  (27.40%) | Q9BRQ8  AIFM2_HUMAN  (26.84%) |
| **NDI1** | Rotenone-insensitive NADH-ubiquinone oxidoreductase, mitochondrial | Alternative NADH-ubiquinone oxidoreductase which catalyzes the oxidation of mitochondrial NADH does not translocate protons across the inner mitochondrial membrane. | GSTUMT00010949001 | Protein ID: 1078657  (90%) | BC1G_07512  (68%) | AN1094  (72%) | NCU11397  (66%) | MGG_06276  (66%) | YML120C  **NDI1**  (41%) | CCX05456.1  U4KWF9 U4KWF9_PYROM  (73%) | F2Z699 NDH2_YARLI  **NDH2**  (60.85%) | Q9BRQ8  AIFM2_HUMAN  22.80% |
| **NUC1** | Mitochondrial nuclease | Major mitochondrial nuclease, has roles in mitochondrial recombination, apoptosis and maintenance of polyploidy | GSTUMT00010203001 | Protein ID: 1011301  (88%) | BC1G_10418  (63%) | AN2185  (68%) | NCU00030  (65%) | MGG_05324  (59%) | YJL208C  **NUC1**  (60%) | CCX06359.1  U4L886 U4L886_PYROM  (66%) | P08466 NUC1_YEAST  **NUC1**  (60.00%) | Q14249 NUCG_HUMAN  (39,32%) |
| **KAP123** | Importin subunit beta-4 | Kap123p, a protein involved in nuclear import, is required for Nuc1p-mediated death | GSTUMT00007010001 | Protein ID: 539593  (90,3%) | BC1G_06016  (48%) | AN2120  (45%) | NCU02357  (23%) | MGG_05869  (37%) | YER110C  **KAP123**  (31%) | CCX30995.1  U4LNT6 U4LNT6_PYROM  (55%) | O60100  IMB4_SCHPO  **kap123**  (37.05%) | Q8TEX9  IPO4_HUMAN (24.56%) |
| **pi038** | Deoxyribonuclease Tat-D | Has both endo- and exonuclease activities. May have a role in apoptosis. | GSTUMT00003965001 | Protein ID: 1013991  (92,2%) | BC1G_12264  (50%) | AN4425  (58%) | NCU03538  (45%) | MGG_06999  (44%) | YBL055C  **YBL055C**  (42,43%) | XP_001214707.1  Q0CLA5 Q0CLA5_ASPTN  (61.00%) | Q9UUF1  YNF8_SCHPO  **pi038**  (50.80%) | Q6P1N9  TATD1_HUMAN  (43.43%) |
| **LAM1** | Membrane-anchored lipid-binding protein LAM1 | Involved in mitochondrial fragmentation during programmed cell death. | GSTUMT00005689001 | Protein ID: 1103683  (86%) | BC1G_07330  (44%) | AN4066  (40%) | NCU00495  (38%) | MGG_08887  (39%) | YHR155W  **LAM1**  (26%) | CCX34380.1  U4LR20 U4LR20_PYROM  (46.00%) | P38851 LAM1_YEAST  **LAM1**  (26.00%) | Q96P50 ACAP3_HUMAN  (20.11%) |
| **YSP2** | Membrane-anchored lipid-binding protein YSP2 | Involved in induction of programmed cell death in response to reactive oxygen species (ROS) | GSTUMT00008310001 | Protein ID: 1060699  (80%) | BC1G_01212  (44%) | AN2215  (46%) | NCU06999  (43%) | MGG_11211  (41%) | YDR326C  **YSP2**  (37%) | CCX29576.1  U4LQJ3 U4LQJ3_PYROM  (42%) | Q06681 YSP2_YEAST  **YSP2**  (37%) | O95759 TBCD8_HUMAN  (17.60%) |
| **STE20** | Serine/threonine-protein kinase STE20 | MAP4K component of the MAPK pathway required for the mating pheromone response, haploid invasive growth and diploid pseudohyphal development. Upon exposure to an apoptotic stimulus, the histone deacetylase Hos3p deacetylates K11 and allows Ste20p to phosphorylate S10, required for apoptotic cell death. | GSTUMT00006969001 (TmelSte20) | Protein ID: 1099152  (88%) | BC1G_05000  (55%) | AN2067  (81%) | NCU03894  (52%) | MGG_06320  (54%) | YHL007C  **STE20**  (49%) | CCX31656.1  U4LUH3 U4LUH3_PYROM  (60%) | Q2VWQ3 STE20_TALMA  **pakA**  (56.38%) | Q13043 STK4_HUMAN  (40.75%) |
| **HOS3** | Histone deacetylase HOS3 | Upon exposure to an apoptotic stimulus, the histone deacetylase Hos3p deacetylates K11 and allows Ste20p to phosphorylate S10, required for apoptotic cell death | GSTUMT00001858001 (TmelHos3) | Protein ID: 843932  (84%) | BC1G_03436  (41%) | AN7019  (36%) | NCU07018  (37%) | MGG_06043  (39%) | YPL116W  **HOS3**  (43%) | CCX34740.1  U4LS10 U4LS10_PYROM  (52,21%) | Q02959  HOS3_YEAST  **HOS3**  (36,15%) | Q8WUI4  HDAC7_HUMAN (22,86%) |
| **LSM1** | Sm-like protein LSm1 | Lsm (Like Sm) protein; forms heteroheptameric complex (with Lsm2p, Lsm3p, Lsm4p, Lsm5p, Lsm6p, and Lsm7p) involved in degradation of cytoplasmic mRNAs; the truncated form of LSM4, leads to an accumulation of mRNAs and the phenotypic markers of apoptosis. | GSTUMT00001151001 | Protein ID: 965788  (90%) | BC1G_15358  (44%) | AN6199  (40%) | ------------------- | --------------------- | YJL124C  **LSM1**  (32.79%) | CCX06587.1 U4KX59 U4KX59_PYROM (64.00%) | P87173 LSM1_SCHPO  **lsm1**  (37.50%) | O15116 LSM1_HUMAN  (36.84%) |
| **LSM4** | Sm-like protein LSm4 | Component of LSm protein complexes, which are involved in RNA processing and may function in a chaperone-like manner. | GSTUMT00005366001 | Protein ID: 971986  (91,9%) | BC1G_06649  (83%) | AN10781  (83%) | NCU07683  (79%) | MGG_01509  (60%) | YER112W  **LSM4**  (46%) | XP_018189849.1  A0A165I2W2 A0A165I2W2_9PEZI  (87%) | Q9Y4Z0 LSM4_HUMAN  **LSM4**  (65.45%) | Q9Y4Z0 LSM4_HUMAN  (65.45%) |
| **DCP1** | mRNA-decapping enzyme-like protein1 | Mutations in the yeast mRNA-decapping pathway, such as those in the LSM1, LSM6, DCP1 and DCP2 genes, show increased mRNA stability and undergo apoptosis | GSTUMT00006552001 | Protein ID: 1191602  (91 %) | BC1G_11753  (33%) | AN7746  (36%) | --------------------- | MGG_05522  (27%) | Q12517 DCP1_YEAST  **DCP1**  (21.68%) | XP_016219270.1  A0A0D1Y302 A0A0D1Y302_9PEZI  (36%) | Q9P805  DCP1_SCHPO  **dcp1**  (25.98%) | Q8IZD4  DCP1B_HUMAN (22.78%) |
| **DCP2** | m7GpppN-mRNA hydrolase | Catalytic component of the decapping complex necessary for the degradation of mRNAs. Blocks autophagy in nutrient-rich conditions by repressing the expression of ATG-related genes through degration of their transcripts | GSTUMT00010106001 | Protein ID: 1113737  (80%) | BC1G_12693  (45%) | AN10010  (51%) | NCU07889  (50%) | MGG_06410  (58%) | YNL118C  **DCP2**  (37%) | XP_018192231.1  A0A165JV92 A0A165JV92_9PEZI  (50%) | O13828  DCP2_SCHPO  **dcp2**  (37.02%) | Q8IU60  DCP2_HUMAN (31.01%) |
| **MCD1** | Sister chromatid cohesion protein 1 | Cleavable component of the cohesin complex, involved in chromosome cohesion during cell cycle. | GSTUMT00006767001 | Protein ID: 895970  (89%) | BC1G_02757  (60%) | AN7465  (38%) | NCU03291  (38%) | MGG_09891  (38%) | YDL003W  **MCD1**  (30%) | XP_011116389.1 S8A357 S8A357_DACHA  (43%) | P30776 RAD21_SCHPO  **rad21**  (36.17%) | O60216 RAD21_HUMAN  (27.08%) |
| **FYV10** | Protein FYV10 | Involved in the proteasome-dependent degradation of fructose-1,6-bisphosphatase and required for survival upon exposure to K1 killer toxin. | GSTUMT00005239001 | Protein ID: 720269  (96%) | BC1G_07372  (53%) | AN8850  (57%) | NCU09000  (48%) | MGG_01665  (46%) | YIL097W  **FYV10**  (26%) | \|  \| \| --- \|   OCK98049.1  (63%) | Q1DTI6  FYV10_COCIM  **FYV10**  (58.90%) | Q7L5Y9  MAEA_HUMAN (36.65%) |
| **SNO1** | Pyridoxal 5'-phosphate synthase subunit SNO1 | Catalyzes the hydrolysis of glutamine to glutamate and ammonia as part of the biosynthesis of pyridoxal 5'-phosphate. The resulting ammonia molecule is channeled to the active site of a SNZ isoform.Curated | GSTUMT00000394001 | Protein ID: 1098308  (91%) | BC1G_10494  (43%) | AN6141  (50%) | NCU06549  (52%) | MGG_05981  (51%) | YMR095C  **SNO1**  (43%) | OGE52824.1  A0A1F5LHZ4 A0A1F5LHZ4_9EURO  (55%) | Q8LAD0  PDX2_ARATH  **PDX2**  (44.71%) | _______________ |
| **RNY1** | Ribonuclease T2-like | Rnase which modulates cell survival under stress conditions. Released from the vacuole to the cytoplasm during stress to promote tRNA and rRNA cleavage and to activate separately a downstream pathway that promotes cell death. | GSTUMT00000865001 | Protein ID: 1024380  (90%) | ________ | AN4874 (36%) | _________ | MGG_05342  (48%) | Q02933 (RNY1_YEAST)  **RNY1**  (31.20%) | KMK55554.1  A0A0J5PJV8 A0A0J5PJV8_ASPFM  (50.00%) | P10281 RNT2_ASPOR  **rntB**  (45.05%) | O00584  RNT2_HUMAN (20.95%) |
| **WHI2** | Growth regulation protein | Plays a role in the coordination of growth and proliferation. Required for entry into G0 phase under conditions of carbon limitation. Involved in the general stress response; acts together with PSR1 to activate stress response element (STRE)-mediated gene expression, possibly through dephosphorylation of MSN2. | GSTUMT00003240001 | Protein ID: 1097740  (93%) | BC1G_00137  (72%) | AN10545  (64%) | NCU10518  (64%) | MGG_11241  (69%) | YOR043W  **WHI2**  (30%) | CCX34109.1  U4LRA7 U4LRA7_PYROM  (75.00%) | Q9P7D3 YOFG_SCHPO  **SPBP4H10.16c**  (37.04%) | Q9Y597  KCTD3_HUMAN (22.76%) |
| **TAH18** | NADPH-dependent diflavin oxidoreductase 1 | Component of the cytosolic iron-sulfur (Fe-S) protein assembly (CIA) machinery. Transfers electrons from NADPH to the Fe-S cluster of DRE2. Positively controls H2O(2)-induced cell death. | GSTUMT00009232001 | Protein ID: 1062451  (84%) | BC1G_01186  (40%) | AN10283  (42%) | NCU09727 (32%) | MGG_01747  (33%) | YPR048W  **TAH18**  (33%) | XP_003300309.1  E3RRQ4 E3RRQ4_PYRTT  (46%) | Q4WU59  NDOR1_ASPFU  **tah18**  (44.50%) | Q9UHB4  NDOR1_HUMAN  (39.10%) |
| **DRE2** | Fe-S cluster assembly protein DRE2 | Component of the cytosolic iron-sulfur (Fe-S) protein assembly (CIA) machinery. Electrons are transferred to the Fe-S cluster from NADPH via the FAD- and FMN-containing protein TAH18. Has anti-apoptotic effects in the cell. Involved in negative control of H2O(2)-induced cell death. | GSTUMT00010409001 | Protein ID: 989706  (83,23) | BC1G_10618  (41%) | AN8485  (38%) | NCU04315  (42%) | MGG_00776  (41%) | YKR071C  **DRE2**  (48%) | XP_011107973.1  S8AM40 S8AM40_DACHA  (47%) | A7EYZ4  DRE2_SCLS1  **dre2**  (48.06%) | Q6FI81  CPIN1_HUMAN (29.77%) |
| **CPR3** | Peptidyl-prolyl cis-trans isomerase C, mitochondrial | PPIases accelerate the folding of proteins. It catalyzes the cis-trans isomerization of proline imidic peptide bonds in oligopeptides. This isozyme is required for growth on lactate at high temperature. | GSTUMT00008859001 (Tmelcyp3) | Protein ID: 1025809  (91%) | BC1G_01528  (67%) | AN4583  (58%) | NCU02614  (64%) | MGG_11257  (62%) | YML078W  **CPR3**  (50%) | CCX16326.1  U4LB12 U4LB12_PYROM  (79%) | Q2TZ33  PPIH_ASPOR  **cyp3**  (66.09%) | O43447  PPIH_HUMAN (60.47%) |
| **PHO36** | ADIPOR-like receptor IZH2 | Plasma membrane protein involved in zinc homeostasis and osmotin-induced apoptosis. | GSTUMT00001471001 | Protin ID: 1047964  (86,3%) | BC1G_04039  (51%) | AN4932  (53%) | NCU04987  (45%) | MGG_05072  (47%) | YOL002C  **PHO36**  (41%) | XP_018061864.1  A0A132B685 A0A132B685_9HELO  (57%) | Q09749  ADRL_SCHPO  **SPBC12C2.09c**  (41.47%) | Q96A54  ADR1_HUMAN (37.19%) |
| **ISC1** | Inositol phosphosphingolipids phospholipase C | Responsible for the hydrolysis of the phosphosphingolipids (IPS), inositol phosphorylceramide (IPC), mannosylinositol phosphorylceramide (MIPC), and mannosyldiinositol phosphorylceramide (M(IP)2C). | GSTUMT00008240001(TmelPLC) | Protein ID: 1089044  (87,6%) | BC1G_06680  (47%) | AN3927  (46%) | NCU03243  (42%) | MGG_04474  (41%) | YER019W  **ISC1**  (32%) | CCX33155.1  U4LTV8 U4LTV8_PYROM  (70%) | O74369  CSS1_SCHPO  **css1**  (42.45%) | O60906  NSMA_HUMAN (28.32%) |
| **ESP1** | Separin | .Caspase-like protease, which plays a central role in the chromosome segregation by cleaving the MCD1/SCC1 subunit of the cohesin complex at the onset of anaphase. | GSTUMT00009703001  (Tmelesp1) | Protein ID: 1130343  (88%) | BC1G_04716  (31%) | AN8783  (31%) | NCU00205  (34%) | MGG_06245  (30%) | YGR098C  **ESP1**  (23.15%) | [CCX30537.1](https://www.ncbi.nlm.nih.gov/protein/549051856?report=genbank&log$=prottop&blast_rank=2&RID=ANYF3ENB015)  U4LG69 U4LG69_PYROM  (44%) | P33144  BIMB_EMENI  **bimB**  (31.88%) | Q14674  ESPL1_HUMAN (21.09%) |
| **YCA1** | Metacaspase-1 | Mediates cell death (apoptosis) triggered by oxygen stress, salt stress or chronological aging. Regulated cell death can prevent a release of toxic cellular components, thus avoiding necrotic collapse of the colony, and can also provide nutrients for healthy cells. Therefore, regulated cell death in yeast colonies can be as important for their development as are apoptosis and related processes that occur within metazoa. Promotes the removal of insoluble protein aggregates during normal growth. | GSTUMT00007513001 | Protein ID: 1033821  (93 %) | BC1G_11354  (68%) | AN5712  (67%) | NCU02400  (63%) | MGG_04926  (68%) | YOR197W  **YCA1**  (57%) | CCX33246.1  U4LW63 U4LW63_PYROM  (83%) | Q0CTN3  MCA1A_ASPTN  **casA**  (69.55%) | P55210  CASP7_HUMAN (22.55%) |
| **CDC48** | Cell division control protein 48 | Involved in spindle disassembly, degradation of ubiquitinated proteins and protein export from the endoplasmic reticulum to the cytoplasm. | GSTUMT00010158001 | Protein ID: 1057556  (99 %) | BC1G_07510  (90%) | AN7254  (87%) | NCU00018  (86%) | MGG_05193  (84%) | YDL126C  **CDC48**  (75%) | CCX34713.1  U4LY19 U4LY19_PYROM  (91%) | Q5AWS6  CDC48_EMENI  **cdc48**  (86.40%) | P55072  TERA_HUMAN (72.86%) |
| **cdc18** | Cell division control protein 18 | Part of the checkpoint control that prevents mitosis from occurring until S phase is completed. | GSTUMT00009908001 | Protein ID: 1116483  (80,7%) | BC1G_00397  (42%) | AN5676  (39%) | NCU02776  (43%) | MGG_01614  (44%) | YJL194W  **CDC6**  (29%) | CZR50680.1  (43%) | P41411  CDC18_SCHPO  **cdc18**  (37.04%) | Q99741  CDC6_HUMAN (30.14%) |
| **MHF1** | Centromere protein S | dsDNA-binding component of a FANCM-MHF complex involved in DNA damage repair and genome maintenance | GSTUMT00003490001 | Protein ID:890740  (95,2%) | BC1G_00715  (65%) | AN0598  (70%) | NCU03629  (64%) | __________ | YOL086W-A  **MHF1**  (34%) | XP_001560687.1  BC1G_00715  (65%) | Q9D084  CENPS_MOUSE  **Apitd1**  (38.02%) | Q8N2Z9  CENPS_HUMAN (35.25%) |
| **MM1** | Translationally-controlled tumor protein homolog | Involved in protein synthesis. Involved in microtubule stabilization | GSTUMT00008989001 | Protein ID: 1098415  (72,2%) | __________ | AN0641  (28%) | NCU06464  (42%) | MGG_06249  (53%) | YKL056C  **MM1**  (38%) | XP_015468899.1  A0A0V1Q2S4 A0A0V1Q2S4_9ASCO  (44%) | Q6C4G1  TCTP_YARLI  **YALI0E27071g**  (52.10%) | P13693  TCTP_HUMAN (38.10%) |
| **NAM8** | Protein NAM8 | Acts as a suppressor of mitochondrial splicing deficiencies when overexpressed. | GSTUMT00001670001 (TmelNam8_like) | Protein ID: 333343  (94%) | BC1G_12954  (78%) | AN9090  (76%) | NCU00768  (73%) | MGG_08741  (71%) | YHR086W  **NAM8**  (42%) | CCX05517.1  U4KVY9 U4KVY9_PYROM  (74%) | O13759 CSX1_SCHPO  **csx1**  (50.25%) | Q9NX07 TSAP1_HUMAN  (38.06%) |
| **ATG1** | Serine/threonine-protein kinase ATG1 | Serine/threonine protein kinase involved in the cytoplasm to vacuole transport (Cvt) and found to be essential in autophagy, where it is required for the formation of autophagosomes. Involved in the clearance of protein aggregates which cannot be efficiently cleared by the proteasome. Plays a key role in ATG9 and ATG23 cycling through the pre-autophagosomal structure and is necessary to promote ATG18 binding to ATG9 through phosphorylation of ATG9. | GSTUMT00009922001 | Protein ID: 1114881 (85,6%) | BC1G_05432 (41%) | AN1632 (42%) | NCU00188 (42%) | MGG_06393 (48%) | YGL180W  **ATG1**  (43%) | XP_001271797.1  A1CHL6  ATG1_ASPCL  (45%) | A1CHL6 ATG1_ASPCL  **atg1**  (45%) | Q6PHR2 ULK3_HUMAN  (31.41) |
| **ATG2** | Autophagy-related protein 2 | Required for cytoplasm to vacuole transport (Cvt) vesicles and autophagosomes completion and in peroxisome degradation. | GSTUMT00007150001 | Protein ID: 332348  (84%) | BC1G_09010  (37%) | AN5491  (39%) | NCU08926  (35%) | MGG_05998  (35%) | YNL242W  **ATG2**  (28%) | SLM36849.1  A0A1W5D1T9 A0A1W5D1T9_9LECA  (42%) | A1CUF9 ATG2_ASPCL  **atg2**  (37.59%) | Q96BY7 ATG2B_HUMAN  (20.43%) |
| **ATG3** | Autophagy-related protein 3 | E2 conjugating enzyme required for the cytoplasm to vacuole transport (Cvt) and autophagy. Responsible for the E2-like covalent binding of phosphatidylethanolamine to the C-terminal Gly of ATG8. The ATG12-ATG5 conjugate plays a role of an E3 and promotes the transfer of ATG8 from ATG3 to phosphatidylethanolamine (PE). | GSTUMT00004299001 | Protein ID: 1024088  (74,6%) | BC1G_08793  (32%) | AN11004  (55%) | NCU01955  (33%) | MGG_02959  (32%) | YNR007C  **ATG3**  (44%) | CDO57435.1  A0A0J9XIN4 A0A0J9XIN4_GEOCN  (38%) | Q2H427 (ATG3_CHAGB)  **ATG3**  (44.5%) | Q9NT62 ATG3_HUMAN  (32.43%) |
| **ATG4** | Cysteine protease ATG4 | Cysteine protease required for cleaves the C-terminal amino acid of ATG8 to reveal a C-terminal glycine. ATG8 ubiquitin-like activity requires the exposure of the glycine at the C-terminus for its conjugation to phosphatidylethanolamine (PE) and its insertion to membranes, which is necessary for autophagy. | GSTUMT00003763001 | Protein ID: 1091534  (91%) | BC1G_11320  (51%) | AN3470  (53%) | NCU02433  (52%) | MGG_03580  (55%) | YNL223W  **ATG4**  (39%) | XP_011114530.1  S8A8M2 S8A8M2_DACHA  (54%) | A6SDQ3 (ATG4_BOTFB)  **atg4**  (52.83%) | Q8WYN0 (ATG4A_HUMAN)  (29.32%) |
| **ATG5** | Autophagy protein 5 | Involved in cytoplasm to vacuole transport (Cvt) and autophagic vesicle formation. Autophagy is essential for maintenance of amino acid levels and protein synthesis under nitrogen starvation. | GSTUMT00011978001 | Protein ID: 1084923  (95,1%) | BC1G_09893  (33%) | AN5174  (50%) | NCU04662  (35%) | MGG_09262  (36%) | YPL149W  **ATG5**  (25%) | CCX34100.1  U4LQ64 U4LQ64_PYROM  54% | A7KAL6 ATG5_PENRW  **atg5**  (48.24%) | Q9H1Y0 ATG5_HUMAN  (32.7%) |
| **atg6** | Vacuolar protein sorting-associated protein atg6 | Required for cytoplasm to vacuole transport (Cvt), autophagy, nucleophagy, and mitophagy, as a part of the autophagy-specific vps34 PI3-kinase complex I. | GSTUMT00008837001 | Protein ID: 1143563  (93,5%) | BC1G_01844  (52%) | AN10213  (57%) | NCU03122  (49%) | MGG_03694  (47%) | YPL120W  **VPS30**  (30%) | CCX16340.1  U4LC18 U4LC18_PYROM  58% | P87117  BECN1_SCHPO  **atg6**  (38,75%) | Q14457 BECN1_HUMAN  (35.48%) |
| **ATG7** | Ubiquitin-like modifier-activating enzyme ATG7 | E1-like activating enzyme involved in the 2 ubiquitin-like systems required for cytoplasm to vacuole transport (Cvt) and autophagy. Activates ATG12 for its conjugation with ATG5 and ATG8 for its conjugation with phosphatidylethanolamineRequired for selective mitophagy which contributes to regulate mitochondrial quantity and quality by eliminating the mitochondria to a basal level to fulfill cellular energy requirements and preventing excess ROS production. | GSTUMT00005517001 | Protein ID: 889018  (89,7%) | BC1G_09811  (50%) | AN7428  (56%) | NCU06672  (57%) | MGG_07297  (51%) | YHR171W  **ATG7**  (38%) | CCX16203.1  U4LHR5 U4LHR5_PYROM  65% | Q871U2 ATG7_NEUCR  **apg-5**  (60.03%) | O95352 ATG7_HUMAN  (44.11%) |
| **ATG8** | Autophagy-related protein 8 | Ubiquitin-like modifier involved in cytoplasm to vacuole transport (Cvt) vesicles and autophagosomes formation. With ATG4, mediates the delivery of the vesicles and autophagosomes to the vacuole via the microtubule cytoskeleton. The ATG8-PE conjugate mediates tethering between adjacent membranes and stimulates membrane hemifusion, leading to expansion of the autophagosomal membrane during autophagy. | GSTUMT00002234001 (TmelAUT7) | Protein ID: 1144196  (100,0%) | BC1G_02467  (97%) | AN5131  (98%) | NCU01545  (98%) | MGG_01062  (93%) | YBL078C  **ATG8**  (78%) | KOS23102.1  A0A0M9VXJ0 A0A0M9VXJ0_9HYPO  (99%) | Q2UBH5 ATG8_ASPOR  **atg8**  (97.46%) | P60520 GBRL2_HUMAN  (58.97%) |
| **ATG9** | Autophagy-related protein 9 | Involved in autophagy and cytoplasm to vacuole transport (Cvt) vesicle formation. Plays a key role in the organization of the preautophagosomal structure/phagophore assembly site (PAS), the nucleating site for formation of the sequestering vesicle. | GSTUMT00000250001 | Protein ID: 1113327  (86,6%) | BC1G_15751  (55%) | AN3734  (41%) | NCU02422  (39%) | MGG_09559  (40%) | YDL149W  **ATG9**  (43%) | EFW13420.1  E9DJG7 E9DJG7_COCPS  (50%) | Q1E6Q3 ATG9_COCIM  **ATG9**  (47.95%) | Q7Z3C6 ATG9A_HUMAN  (27.27%) |
| **atg11** | Autophagy-related protein 11 | Involved in cytoplasm to vacuole transport (Cvt), pexophagy, mitophagy and nucleophagy. Recruits mitochondria for their selective degradation via autophagy (mitophagy) during starvation. Works as scaffold proteins that recruit ATG proteins to the pre-autophagosome (PAS), the site of vesicle/autophagosome formation. Required for the Cvt vesicles completion | GSTUMT00000963001 | Protein ID: 1053020  (86%) | BC1G_03242  (30%) | AN2887  (31%) | NCU09998  (29%) | MGG_04486  (28%) | --------------- | CCX17165.1  U4LJ69 U4LJ69_PYROM  (42%) | Q4WY31 ATG11_ASPFU  **atg11**  (33.13%) | P55196 AFAD_HUMAN  (15.63%) |
| **ATG12** | Ubiquitin-like protein ATG12 | Ubiquitin-like protein involved in cytoplasm to vacuole transport (Cvt), autophagy vesicles formation, mitophagy, and nucleophagy. Conjugation with ATG5 through a ubiquitin-like conjugating system involving also ATG7 as an E1-like activating enzyme and ATG10 as an E2-like conjugating enzyme, is essential for its function. The ATG12-ATG5 conjugate functions as an E3-like enzyme which is required for lipidation of atg8 and atg8 association to the vesicle membranes | GSTUMT00006991001 | Protein ID: 1061820  (82,9%) | BC1G_14655  (56%) | AN1760  (50%) | NCU10049  (41%) | MGG_00598  (53%) | YBR217W  **ATG12**  (41%) | CCX04868.1  U4KUB6 U4KUB6_PYROM  (59%) | Q2UMW6 ATG12_ASPOR  **atg12**  (48.98%) | O94817 ATG12_HUMAN  (26.12%) |
| **ATG13** | Autophagy-related protein 13 | Activates the atg1 kinase in a nutritional condition dependent manner through the TOR pathway, leading to autophagy. Also involved in cytoplasm to vacuole transport (Cvt) and more specifically in Cvt vesicle formation. Seems to play a role in the switching machinery regulating the conversion between the Cvt pathway and autophagy. | GSTUMT00007009001 | Protein ID: 1054155  (83,4) | BC1G_15976  (37%) | AN2076  (36%) | NCU04840  (34%) | MGG_00454  (35%) | YPR185W  **ATG13**  (25%) | XP_011127578.1  G1XTE5 G1XTE5_ARTOA  (46%) | A7F7B2 ATG13_SCLS1  **atg13**  (39.72%) | O75143 ATG13_HUMAN  (18.42%) |
| **ATG15** | Putative lipase ATG15 | Lipase which is essential for lysis of subvacuolar cytoplasm to vacuole targeted bodies and intravacuolar autophagic bodies. Involved in the lysis of intravacuolar multivesicular body (MVB) vesicles. | GSTUMT00010596001 | Protein ID: 1191935  (84,6%) | BC1G_07555  (46%) | AN5919  (50%) | NCU06436  (49%) | MGG_12828  (60%) | YCR068W  **ATG15**  (40%) | CCX33132.1  U4LNG1 U4LNG1_PYROM  (57%) | A6REI4 ATG15_AJECN  **ATG15**  (50.44%) | ---------------------- |
| **ATG16** | Autophagy protein 16 | Stabilizes the ATG5-ATG12 conjugate which is necessary for autophagy. The ATG5-ATG12/ATG16 complex is required for efficient promotion of ATG8-conjugation to phosphatidylethanolamine and ATG8 localization to the pre-autophagosomal structure (PAS). | GSTUMT00007722001 | Protein ID: 1194653  (89,6%) | BC1G_07590  (28%) | AN0090  (29%) | __________ | MGG_05255  (27%) | YMR159C ATG16 (25%) | XP_018192144.1  A0A165JT53 A0A165JT53_9PEZI  (36%) | Q6C4Y4 ATG16_YARLI  **ATG16**  (32.58%) | Q676U5 A16L1_HUMAN  (21.88%) |
| **ATG17** | Autophagy-related protein 17 | Autophagy-specific protein that functions in response to autophagy-inducing signals as a scaffold to recruit other ATG proteins to organize pre-autophagosomal structure (PAS) formation. | GSTUMT00000397001 | Protein ID: 897679  (91,5%) | BC1G_11270  (30%) | AN6360  (31%) | NCU08766  (30%) | MGG_07667  (28%) | YLR423C  **ATG17**  (23%) | XP_011119363.1  G1X4F1 G1X4F1_ARTOA  (39%) | A2QCV0 ATG17_ASPNC  **atg17**  (35.17%) | P78396 CCNA1_HUMAN  (21.87%) |
| **ATG18** | Autophagy-related protein 18 | Necessary for proper vacuole morphology. Plays an important role in osmotically-induced vacuole fragmentation. Required for cytoplasm to vacuole transport (Cvt) vesicle formation, pexophagy and starvation-induced autophagy. | GSTUMT00001405001 | Protein ID: 1097242  (96,7%) | BC1G_12821  (52%) | AN0127  (59%) | NCU03441  (53%) | MGG_03139  (55%) | YFR021W  **ATG18**  (39%) | CCX32475.1  U4LSV9 U4LSV9_PYROM  (74%) | Q1DKJ3 ATG18_COCIM  **ATG18**  (63.64%) | Q5MNZ9 WIPI1_HUMAN  (40.46%) |
| **ATG20** | Autophagy-related protein 20 | Required for cytoplasm to vacuole transport (Cvt), pexophagy and mitophagy. | GSTUMT00009504001 | Protein ID: 1164213  (87,9) | BC1G_14116  (51%) | AN6351  (56%) | NCU06259  (51%) | MGG_12832  (51%) | YDL113C  **ATG20**  (32%) | CCX34523.1  U4LXM5 U4LXM5_PYROM  (60%) | Q5AZC9 SNX41_EMENI  **snx41**  (56.57%) | Q9UNH6 SNX7_HUMAN  (22.99%) |
| **ATG22** | Autophagy-related protein 22 | Vacuolar effluxer which mediate the efflux of amino acids resulting from autophagic degradation. | GSTUMT00006789001 | Protein ID: 1087266  (89,2%) | BC1G_07239  (59%) | AN7437  (54%) | NCU07504  (51%) | MGG_09904  (54%) | YCL038C  **ATG22**  (34%) | CCX30457.1  U4LFZ1 U4LFZ1_PYROM  70% | A7EXE6 AT222_SCLS1  **atg22-2**  (58.63%) | ------------------ |
| **ypt-1** | GTP-binding protein ypt1 | The small GTPases Rab are key regulators of intracellular membrane trafficking, from the formation of transport vesicles to their fusion with membranes. Ypt-1 plays a role in the initial events of the autophagic vacuole development which take place at specialized regions of the endoplasmic reticulum. | GSTUMT00002466001 | Protein ID: 1043812  (98,9%) | BC1G_02688  (92%) | AN4281  (94%) | NCU08477  (89%) | MGG_06962  (89%) | YFL038C  **YPT1**  (71%) | XP_007776948.1  R7YJ47 R7YJ47_CONA1  (94%) | P33723 YPT1_NEUCR  **ypt-1**  (89.55%) | P62820 RAB1A_HUMAN  (80.90%) |
| **YPT31** | RAB11A, member RAS oncogene family. | Required for protein transport in the secretory pathway. Probably involved in regulation of secretory vesicle formation at the trans-Golgi compartment. Plays a role in autophagy. | GSTUMT00009481001 | Protein ID: 1063008  (94%) | BC1G_09346  (83%) | AN0347  (82%) | NCU01523  (81%) | MGG_01079  (75%) | YER031C  **YPT31**  (63%) | XP_007757423.1 W9W7V0 W9W7V0_9EURO  (85%) | Q5ZJN2 RB11A_CHICK  **RAB11A**  (78.67%) | P62491 RB11A_HUMAN  (78.67%) |
| **MMS2** | Ubiquitin-conjugating enzyme spm2 | Has a role in the DNA error-free postreplication repair (PRR) pathway. Lacks catalytic activity by itself. The UBC13/MMS2 heterodimer catalyzes the synthesis of non-canonical poly-ubiquitin chains that are linked through 'Lys-63'. | GSTUMT00003288001 | Protein ID: 1051487  (83,5%) | BC1G_00036  (68%) | AN3644  (69%) | NCU10477  (50%) | MGG_06562  (45%) | YGL087C  **MMS2**  (62%) | XP_013946438.1  G9NN00 G9NN00_HYPAI  (71%) | O74983 MMS2_SCHPO  **spm2**  (63.77%) | Q13404  UB2V1_HUMAN  (47.48%) |
| **UBC13** | Ubiquitin-conjugating enzyme E2 13 | Has a role in the DNA error-free postreplication repair (PRR) pathway. The UBC13/MMS2 heterodimer catalyzes the synthesis of non-canonical poly-ubiquitin chains that are linked through 'Lys-63' | GSTUMT00004713001 | Protein ID: 1055328  (97,3%) | BC1G_11203  (91%) | AN8702  (90%) | NCU02113  (85%) | MGG_02568  (86%) | YDR092W  **UBC13**  (73.83%) | XP_747835.1  Q4WCU1 Q4WCU1_ASPFU  (93%) | P52490 UBC13_YEAST  **UBC13**  (73.83%) | P61088 UBE2N_HUMAN  (72.48%) |
| **usp22** | Ubiquitin carboxyl-terminal hydrolase 22 | Histone deubiquitinating component of the transcription regulatory histone acetylation (HAT) complex SAGA. Catalyzes the deubiquitination of both histones H2A and H2B, thereby acting as a coactivator. | GSTUMT00007445001 | Protein ID:1023174  (84,5%) | BC1G_16159  (35%) | AN3711  (42%) | NCU02375  (40%) | MGG_03527  (42%) | YNL186W  **UBP10**  (25%) | CCX16461.1  U4LC96 U4LC96_PYROM  (62%) | A6H8I0 UBP22_DANRE  **usp22**  (37.37%) | Q9UPT9 UBP22_HUMAN  (36.42%) |
| **ASF1** | Histone chaperone ASF1 | Histone chaperone that facilitates histone deposition and histone exchange and removal during nucleosome assembly and disassembly. | GSTUMT00007519001 | Protein ID: 896629  (96,8%) | BC1G_06943  (71%) | AN4891  (70%) | NCU09436  (63%) | MGG_14445  (56%) | YJL115W  **ASF1**  (51.69%) | XP_010756467.1  C1G1H8 C1G1H8_PARBD  (70%) | Q5B3I9 ASF1_EMENI  **asf1**  (63.70%) | Q9Y294 ASF1A_HUMAN  (45.13%) |
| **RAS2** | Ras-like protein 2 | Ras proteins bind GDP/GTP and possess intrinsic GTPase activity. Hyper-activation of the Ras protein Ras2p, part of a signaling pathway upstream from PKA activation, increases apoptosis | GSTUMT00002293001 (TmelRAS2) | Protein ID:1189643  (88,6%) | BC1G_04437  (81%) | AN5832  (64%) | NCU03616  (71%) | MGG_09499  (69%) | YNL098C  **RAS2**  (56%) | KJZ76339.1  A0A0F8A625 A0A0F8A625_9HYPO  (77%) | Q01387 RAS2_NEUCR  **ras-2**  (71%) | P01111 RASN_HUMAN  (53.48%) |
| **STM1** | Suppressor protein STM1 | Binds specifically G4 quadruplex and purine motif triplex nucleic acid structures. These structures may be present at telomeres or in rRNAs. Acts with CDC13 to control telomere length homeostasis. Involved in the control of the apoptosis-like cell death. | GSTUMT00000007001 | Protein ID:1054260  (78,4%) | BC1G_15577  (39%) | AN10614  (38%) | NCU00225  (38%) | _____________ | YLR150W  **STM1**  (32.46%) | EWC44050.1  W7I5P7 W7I5P7_9PEZI  (43%) | O42914 YBI8_SCHPO  **SPBC16A3.08c**  (34.1’%) | ______________ |
| **DAD1** | Dolichyl-diphosphooligosaccharide--protein glycosyltransferase subunit DAD1 | Essential subunit of the N-oligosaccharyl transferase (OST) complex which catalyzes the transfer of a high mannose oligosaccharide from a lipid-linked oligosaccharide donor to an asparagine residue within an Asn-X-Ser/Thr consensus motif in nascent polypeptide chains. Loss of the DAD1 protein triggers apoptosis. | GSTUMT00010257001 | Protein ID:1112219  (88,3%) | BC1G_00509  (79%) | AN4031  (49%) | NCU09216  (73%) | MGG_05758  (71%) | YOR103C  **OST2**  (49%) | XP_011111431.1  S8BM77 S8BM77_DACHA  (63%) | Q29036  DAD1_PIG  **DAD1**  (49.56%) | P61803 DAD1_HUMAN  (48.67%) |
