# Supplementary material for: The genomic tool-kit of the truffle Tuber melanosporum programmed cell death
Source: Cell Death Discov. 2018 Feb 20;4:32. doi: 10.1038/s41420-017-0019-0 (PMC5841409; doi:10.1038/s41420-017-0019-0)
Supplement: Supplementary file 3 — Supplementary Table S2 [file 41420_2017_19_MOESM3_ESM.pdf]

Table S2- Expression of 67 *T. melanosporum* PCD related genes in various stages (III-VI) of FBs.						
Tuber gene model	***Protein***	***StageIII***	***StageIV***	***StageV***	***StageVI***	***qPCR***
**GSTUMT00004899001**	**Bax inhibitor 1**	**38814**	**31843**	**33056**	**31843**	**x**
GSTUMT00009609001	Mitochondrial phosphate carrier protein	14393	27904	14691	15760	
**GSTUMT00005644001**	**ADP,ATP carrier protein 2**	**32836**	**35706**	**30195**	**42733**	**x**
GSTUMT00003910001	Mitochondrial outer membrane protein porin 1	12278	17835	22131	27435	
**GSTUMT00002150001**	**Mitochondrial fission 1 protein**	**32608**	**26781**	**28312**	**25038**	**x**
GSTUMT00010895001	Mitochondrial division protein 1	13696	14288	17244	14288	
**GSTUMT00001624001**	**Dynamin-related protein DNM1**	**12322**	**16225**	**13628**	**12322**	**x**
GSTUMT00006726001	Protein bir1	480	314	440	460	
**GSTUMT00008284001**	**Pro-apoptotic serine protease NMA111**	**10392**	**9582**	**4363**	**7525**	**x**
**GSTUMT00001651001**	**Apoptosis-inducing factor 1**	**19435**	**15843**	**14694**	**14446**	**x**
**GSTUMT00004637001**	**Apoptosis-inducing factor 2**	**14170**	**5531**	**4413**	**12872**	**x**
GSTUMT00010949001	Rotenone-insensitive NADH-ubiquinone oxidoreductase, mitochondrial	10450	17216	9824	5735	
**GSTUMT00010203001**	**Mitochondrial nuclease**	**5649**	**6593**	**4580**	**11328**	**x**
GSTUMT00007010001	Importin subunit beta-4	869	1326	5482	1326	
GSTUMT00003965001	Deoxyribonuclease Tat-D	5880	3368	8495	6302	
GSTUMT00005689001	Membrane-anchored lipid-binding protein LAM1	2533	4018	2533	1754	
GSTUMT00008310001	Membrane-anchored lipid-binding protein YSP2	690	465	130	163	
**GSTUMT00006969001**	**Serine/threonine-protein kinase STE20**	**7639**	**10821**	**12464**	**10149**	**x**
GSTUMT00001858001	Histone deacetylase HOS3	1132	992	526	369	
GSTUMT00001151001	Sm-like protein LSm1	17300	7129	8085	6534	
GSTUMT00005366001	Sm-like protein LSm4	1150	1311	1853	412	
GSTUMT00006552001	mRNA-decapping enzyme-like protein1	6305	6867	7439	3172	
GSTUMT00010106001	m7GpppN-mRNA hydrolase	24033	26351	18757	13510	
GSTUMT00006767001	Sister chromatid cohesion protein 1	501	231	223	346	
GSTUMT00005239001	Protein FYV10	16063	11953	13084	7607	

GSTUMT00000394001	Pyridoxal 5'-phosphate synthase subunit SNO1	3102	7918	9745	15705	
GSTUMT00000865001	Ribonuclease T2-like	69	50	27	23	
GSTUMT00003240001	Growth regulation protein	10826	12712	10629	12921	
GSTUMT00009232001	NADPH-dependent diflavin oxidoreductase 1	4026	5049	4386	1750	
GSTUMT00010409001	Fe-S cluster assembly protein DRE2	10682	17423	11136	11136	
GSTUMT00008859001	Peptidyl-prolyl cis-trans isomerase C, mitochondrial	7930	11872	13895	17993	
GSTUMT00001471001	ADIPOR-like receptor IZH2	1338	1756	490	1338	
GSTUMT00008240001	Inositol phosphosphingolipids phospholipase C	2110	1365	1774	3577	
GSTUMT00009703001	Separin	241	179	436	510	
**GSTUMT00007513001**	**Metacaspase-1**	**19719**	**22432**	**26148**	**16699**	**x**
**GSTUMT00010158001**	**Cell division control protein 48**	**11374**	**14213**	**10247**	**14324**	**x**
GSTUMT00009908001	Cell division control protein 18	35308	7825	14995	8600	
GSTUMT00003490001	Centromere protein S	10298	10691	14680	11924	
GSTUMT00008989001	Translationally-controlled tumor protein homolog	13116	11311	11711	20734	
GSTUMT00001670001	Protein NAM8	22909	21663	18599	17578	
GSTUMT00009922001	Serine/threonine-protein kinase ATG1	3899	2009	959	1724	
GSTUMT00007150001	Autophagy-related protein 2	5291	1878	721	1251	
GSTUMT00004299001	Autophagy-related protein 3	20371	18591	16513	16745	
GSTUMT00003763001	Cysteine protease ATG4	14090	18726	12033	11278	
GSTUMT00011978001	Autophagy protein 5	9072	9281	12159	9632	
GSTUMT00008837001	Vacuolar protein sorting-associated protein atg6	4151	4343	3388	6878	
GSTUMT00005517001	Ubiquitin-like modifier-activating enzyme ATG7	10188	8466	12597	9816	
**GSTUMT00002234001**	**Autophagy-related protein 8**	**30151**	**30634**	**35649**	**33554**	**x**
GSTUMT00000250001	Autophagy-related protein 9	4564	3474	4772	2261	
GSTUMT00000963001	Autophagy-related protein 11	2966	2468	1178	1185	
GSTUMT00006991001	Ubiquitin-like protein ATG12	3464	7010	9332	6363	
GSTUMT00007009001	Autophagy-related protein 13	605	443	358	282	
GSTUMT00010596001	Putative lipase ATG15	6464	9023	3938	3751	

GSTUMT00007722001	Autophagy protein 16	9929	8160	7745	12812	
GSTUMT00000397001	Autophagy-related protein 17	3647	1671	1194	750	
GSTUMT00001405001	Autophagy-related protein 18	12812	8266	12659	8779	
GSTUMT00009504001	Autophagy-related protein 20	9111	7193	10041	6359	
GSTUMT00006789001	Autophagy-related protein 22	10783	10205	7682	7455	
GSTUMT00002466001	GTP-binding protein ypt1	29494	31344	30108	34665	
GSTUMT00009481001	RAB11A, member RAS oncogene family.	16845	22162	22838	28392	
**GSTUMT00003288001**	**Ubiquitin-conjugating enzyme spm2**	**18508**	**22841**	**26597**	**21197**	**x**
GSTUMT00004713001	Ubiquitin-conjugating enzyme E2 13	16463	19920	19947	23566	
GSTUMT00007445001	Ubiquitin carboxyl-terminal hydrolase 22	8296	5281	3232	5263	
**GSTUMT00007519001**	**Histone chaperone ASF1**	**5210**	**6343**	**6172**	**3710**	**x**
GSTUMT00002293001	Ras-like protein 2	19110	16937	10968	19240	
GSTUMT00000007001	Suppressor protein STM1	6452	3571	4270	4154	
GSTUMT00010257001	Dolichyl-diphosphooligosaccharide--protein glycosyltransferase subunit DAD1	33601	35087	33601	33624	
