# Supplementary figures and images for: The genomic tool-kit of the truffle Tuber melanosporum programmed cell death
Source: Cell Death Discov. 2018 Feb 20;4:32. doi: 10.1038/s41420-017-0019-0 (PMC5841409; doi:10.1038/s41420-017-0019-0)

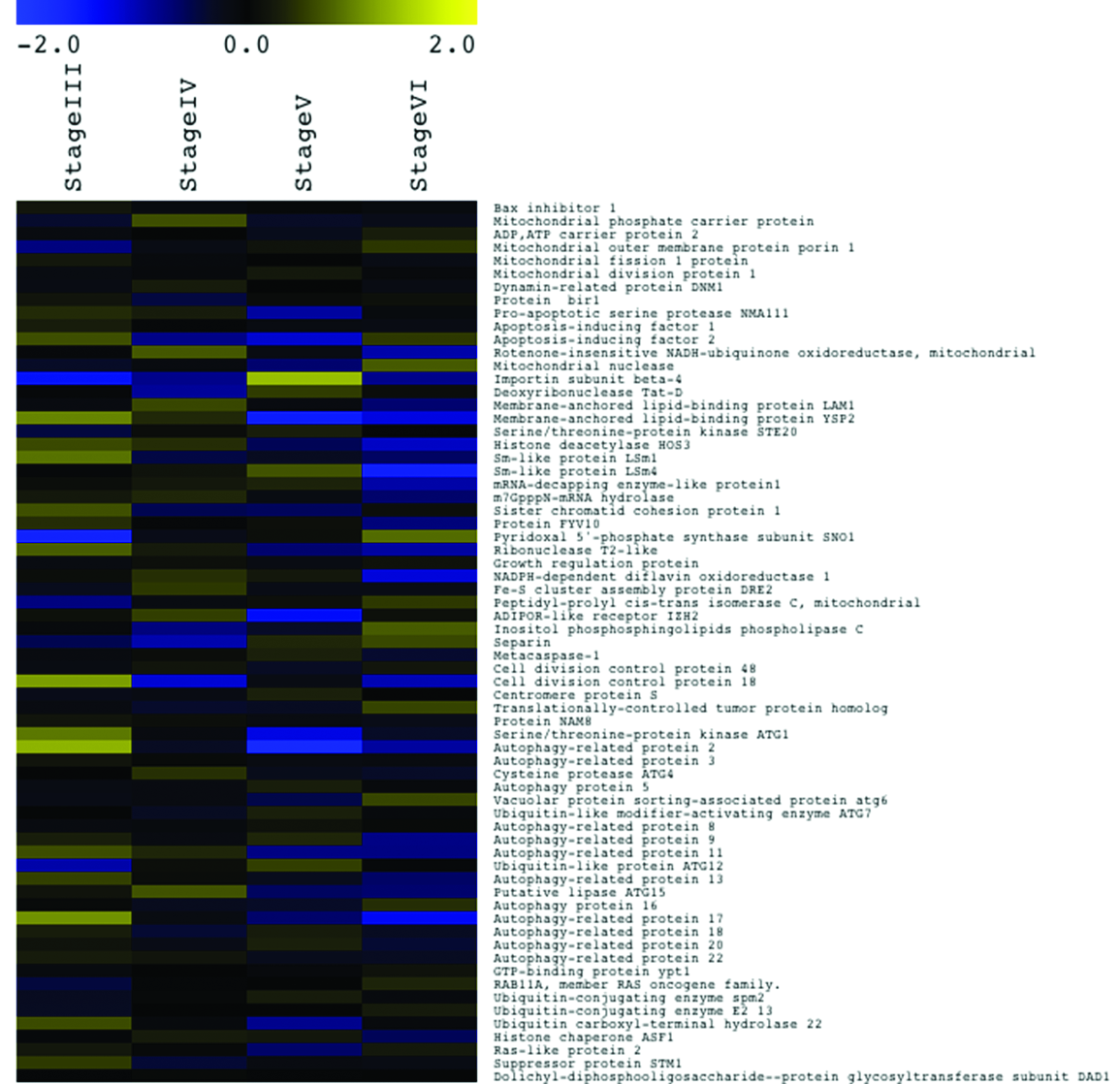

Supplement: Supplementary file 4 — Supplementary Figure S1 [file 41420_2017_19_MOESM4_ESM.tif]

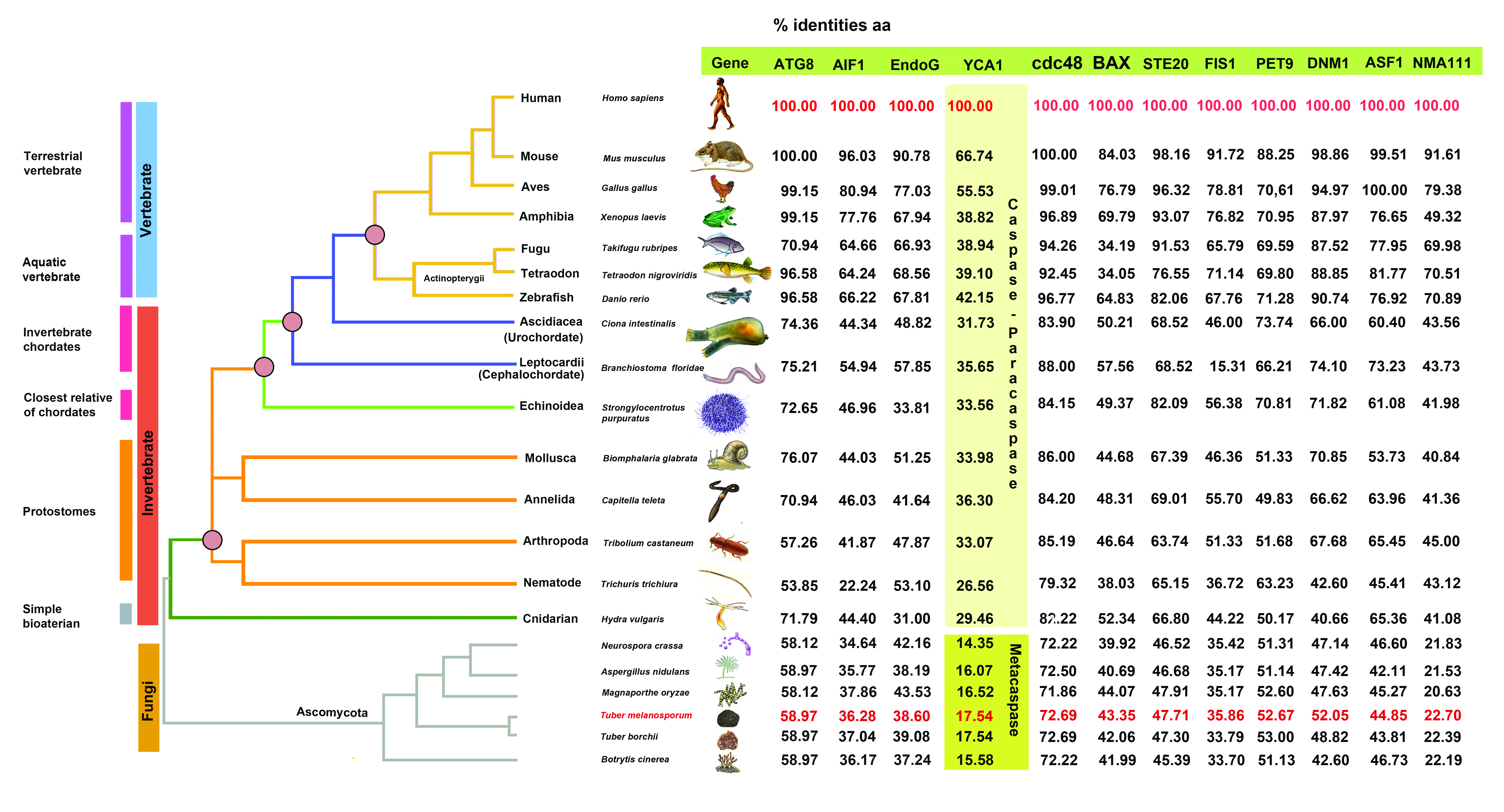

Supplement: Supplementary file 5 — Supplementary Figure S2 [file 41420_2017_19_MOESM5_ESM.tif]
